# A genome-wide genetic screen identifies a novel kDNA replication protein in trypanosomes

**DOI:** 10.1093/nar/gkag493

**Published:** 2026-05-21

**Authors:** Migla Miskinyte, Clirim Jetishi, Ana Kalichava, Alasdair Ivens, Martin Waterfall, Matthew K Gould, Lucy Glover, David Horn, Torsten Ochsenreiter, Achim Schnaufer

**Affiliations:** Institute of Immunology and Infection Research, University of Edinburgh, Edinburgh EH9 3FL, United Kingdom; Institute of Cell Biology, Faculty of Science, University of Bern, Bern 3012, Switzerland; Graduate School for Cellular and Biomedical Sciences, University of Bern, Bern 3012, Switzerland; Institute of Cell Biology, Faculty of Science, University of Bern, Bern 3012, Switzerland; Graduate School for Cellular and Biomedical Sciences, University of Bern, Bern 3012, Switzerland; Institute of Immunology and Infection Research, University of Edinburgh, Edinburgh EH9 3FL, United Kingdom; Institute of Immunology and Infection Research, University of Edinburgh, Edinburgh EH9 3FL, United Kingdom; Institute of Immunology and Infection Research, University of Edinburgh, Edinburgh EH9 3FL, United Kingdom; Faculty of Life Sciences, University of Dundee, Dundee DD1 5EH, United Kingdom; Faculty of Life Sciences, University of Dundee, Dundee DD1 5EH, United Kingdom; Institute of Cell Biology, Faculty of Science, University of Bern, Bern 3012, Switzerland; Institute of Immunology and Infection Research, University of Edinburgh, Edinburgh EH9 3FL, United Kingdom

## Abstract

Mitochondrial DNA of trypanosomatid parasites is organized into a topologically complex structure, named kinetoplast [kinetoplast DNA (kDNA)]. Replication, segregation and expression of kDNA involve an estimated ∼300 proteins, only a fraction of which have been identified and characterized. Here, we report the development of a genetic screen in *Trypanosoma brucei* to identify novel kDNA maintenance factors. Of the 20 highest-ranked genes identified, six are known kDNA maintenance factors. We selected one hit, *Tb927.8.4240*, a gene of previously unknown function, for further experimental characterization. Ultrastructure expansion microscopy using a tagged version of the protein reveals a dynamic localization during the cell cycle. RNA interference-mediated ablation of *Tb927.8.4240* results in the progressive but incomplete loss of kDNA, with only a minor effect on the tripartite attachment complex, suggesting the protein is involved in kDNA replication but not segregation. The growth phenotype of *Tb927.8.4240* ablation is fully rescued in a kDNA-independent genetic background, confirming a specific role in kDNA replication. In summary, we describe a functional genetic screen for the identification of kDNA maintenance factors in trypanosomes, validate one hit as a novel kDNA replication factor, and provide a prioritized hit list as a promising starting point for the future identification of additional factors.

## Graphical Abstract

**Graphical Abstract:**
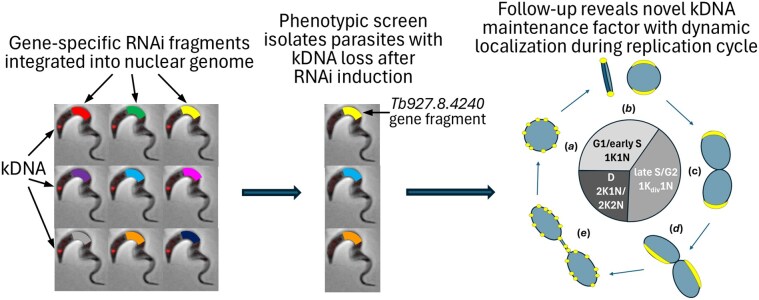

## Introduction

Kinetoplastids are a group of flagellated protists that include the important human and animal pathogens of the genera *Trypanosoma* and *Leishmania* [1]. A hallmark feature of these organisms is the kinetoplast DNA (kDNA), the organellar DNA within their single mitochondrion [2]. Like mitochondrial DNA of other eukaryotes, kDNA encodes subunits of the mitochondrial oxidative phosphorylation (OXPHOS) system and the mitoribosome [3–5]. However, unlike typical mitochondrial DNA, kDNA is arranged in a massive, catenated network comprising 5000–10 000 minicircles and dozens of maxicircles, all physically linked into a planar disk [2, 6]. The kDNA disk itself is physically linked to the flagellum’s basal body by the so-called tripartite attachment complex (TAC), a fibrous structure that crosses both mitochondrial membranes [7]. The intricate kDNA network is not only visually striking when observed with electron or atomic force microscopy [8, 9] but is also essential for the survival and propagation of trypanosomes, as the encoded genes are critical for mitochondrial function and parasite viability [2, 5, 6, 10].

The complexity of the kDNA structure is functionally linked to equally complex mechanisms for mitochondrial gene expression. Out of the 18 messenger RNA (mRNAs) encoded in the maxicircles (20–30 kb in length in *Trypanosoma brucei*), 12 require post-transcriptional editing by insertion and removal of uridines [11]. For some mRNAs this editing is extensive and results in more than doubling of the mRNA length. The precise number of uridines to be inserted or removed, and the location of editing events within the mRNAs, is specified by short guide RNAs (gRNAs) by virtue of their complementarity to the fully edited sequence. The vast majority of gRNAs are encoded by minicircles (∼1 kb in length in *T. brucei*, with 3–4 gRNA genes per minicircle [12]). Hence, the sequence information for most kDNA-encoded genes is split between maxicircles and minicircles, and faithful replication and transfer to daughter cells of both types of molecules during cell division is therefore critical for parasite viability. Indeed, interference with kDNA maintenance is a key part of the mode of action of several anti-trypanosomatid drugs [13, 14].

Unsurprisingly, elucidation of the precise mechanism of kDNA replication and segregation, and of the molecular machineries responsible for these processes, has been a major focus of research, and the current state of knowledge of these processes has been detailed in several reviews (e.g. [2, 15, 16]). Replication and segregation of kDNA are highly regulated, multi-phase processes that occur once per cell cycle and involve the orchestrated action of numerous enzymatic and structural proteins and complexes. Summarized briefly, replication initiates with the release of individual minicircles from the kDNA network (presumably by topoisomerase 2) into the so-called kinetoflagellar zone (KFZ) between kDNA disk and inner mitochondrial membrane, where they undergo unidirectional theta-type replication [17]. The process probably involves DNA polymerase IB and universal minicircle binding protein 1 [18, 19]. The resulting daughter minicircles are then reattached to the network at so-called antipodal sites (APS) at opposite sides of the network periphery, a process that must require precise topological control to prevent entanglement and ensure network integrity [16]. The APS are still poorly defined in composition and structure, but have been reported to contain several components of the kDNA replication machinery, including enzymes required for reattachment of minicircles into the network (topoisomerase 2 [20], possibly MiRF172 [21]), initiation of replication (primase 2) [22], primer removal (helicases PIF1 and PIF5) [23, 24], and gap repair (polymerase β [25], DNA ligase LigK-β [26]). The traditional model for minicircle replication postulates a sorting mechanism that ensures the distribution of the two daughter minicircles to opposite APS [2]. More recently, reexamination of available localization data for kDNA replication factors led to an alternative ‘loose-diploid’ model that envisions two distinct lobes within the kDNA disk, each with an essentially complete set of minicircles that are released from the network laterally, replicated and then reattached to the same lobe [27]. In both models, maxicircles remain attached to the network for replication [28], which likely involves DNA polymerases IC and ID [29, 30].

Following replication, the accurate segregation of the kDNA daughter networks into daughter cells is facilitated by the TAC, which links this process to the duplication and segregation of the basal body during cell division. The molecular architecture and biogenesis of the TAC has been dissected in considerable detail in recent years (reviewed in [15, 16]). TAC consists of three distinct subdomains: the extramitochondrial exclusion zone filaments (EZF) between the basal body and the outer mitochondrial membrane, the intramitochondrial unilateral filaments (ULF) between inner mitochondrial membrane and kDNA, and the differentiated membranes (DM) that bridge EZF and ULF. The DM subdomain is complex and consists of at least four different proteins (pATOM36, TAC40, TAC42, and TAC60) [15]. The EZF appears to consist of a single component, p197, that directly binds the basal body and is linked to the DM via TAC65 [31]. The ULF consists of at least TAC53 [32], p166 [33], and TAC102 [34]; the molecular details of its interaction with the kDNA disk itself remains to be elucidated, but may involve at least two additional proteins that are not part of the TAC itself [35].

Overall, 63 different proteins have to date been reported as ‘kinetoplast-associated’ [36], which includes the kDNA replication factors and TAC components described above, and it was estimated that kDNA repair, replication and segregation may involve around 150 proteins [8]. Important aspects of these processes that remain to be elucidated include the precise structural organization of kDNA replication, including the mechanism of transport of minicircle replication intermediates to the reattachment sites, how topology and size of the network are controlled, and how replication of kDNA and of nuclear DNA are coordinated and integrated into the cell cycle.

The impressive progress that has been made in identifying components of kDNA replication and segregation in trypanosomes has resulted from a combination of bioinformatics, biochemical and imaging approaches, with each providing complementary insights into the molecular and cellular players and processes. In recent years, genome-wide RNA interference (RNAi) screens in trypanosomes that select for phenotypes of interest after gene ablation have become a powerful tool in trypanosome research [37]. An early version of such a screen for the identification of factors involved in kDNA replication and segregation depended on initial isolation of clonal cell lines, followed by individual microscopic analysis of RNAi-induced cells to find cases of kDNA loss or shrinkage [38]. Here, we describe the development of a genome-wide RNAi screen that incorporates isolation of cell populations with depleted kDNA by fluorescence-activated cell sorting (FACS), followed by DNA deep sequencing to identify novel candidate genes with critical roles in kDNA maintenance and its regulation. We demonstrate the functionality of the screen, discuss how it could be further improved in the future, and present experimental characterization of one hit as a novel kDNA maintenance factor with an unusual, dynamic localization during the kDNA replication cycle.

## Materials and methods

### *Trypanosoma brucei* growth and whole-genome RNAi library generation

Bloodstream-form *T. brucei* 2T1^T7^ cells, expressing T7 RNA polymerase (T7RNAP) and tet repressor protein (TetR) for robust inducible expression of RNAi inserts, and resistant to blasticidin and puromycin, were cultured according to the published protocol [39]. Cells were maintained in HMI-11 medium [40], supplemented with 10% fetal calf serum (FCS; Gibco) and 50 μg/ml penicillin/streptomycin (Thermo Fisher Scientific), under a 5% CO_2_ atmosphere, unless otherwise specified. A mutated F_1_F_O_-ATPase γ subunit (γL262P) was introduced into the 2T1^T7^ cell line by transfection with the NotI-digested pEnT6-NEO- γL262P plasmid, as described [41], and selected for resistance to neomycin at a concentration of 1 μg/ml. To confirm replacement of both alleles (γL262P/γL262P genotype), the relevant genomic region from selected transfectants was polymerase chain reaction (PCR)-amplified and sequenced using primers 6 and 141 (Supplementary Table ST1 lists all PCR, quantitative reverse transcriptase-polymerase chain reaction (RT-PCR) and sequencing primers for this study). In contrast to the published procedure for generation of genome-wide RNAi libraries [39], expression of I-SceI meganuclease did not increase recombination and transfection efficiency in our cell lines. To obtain the desired library complexity, a total of 2.8 × 10^9^ 2T1^T7^-γL262P cells were transfected with 664 μg of the linearized pZJM RNAi library, using a total of 54 independent transfections (∼5.1 × 10^7^ cells and 12 μg of DNA per transfection). Following selection, transfected cells were grown in 300 ml of HMI-11 medium supplemented with 2 μg/ml phleomycin. After 5 days, cells were collected by centrifugation and resuspended in fresh HMI-11 medium with phleomycin, with selection continuing for an additional 4 days.

### Fluorescence-activated cell sorting

FACS was performed using a FACSAria cell sorter (BD Biosciences). *Trypanosoma brucei* cells were grown under standard conditions at 37°C with 5% CO_2_, using HMI-11 medium with 10% FCS and antibiotics. To ensure cell vitality, the cultures were maintained in the exponential growth phase, not exceeding a density of 1 × 10^6^ cells/ml. During experimental procedures, cells were centrifuged at 1200 × *g* for 5 min and then resuspended in Creek’s Minimal Media (CMM) [42] with an additional 10% FCS, followed by a recovery period of 2–3 h at a lowered density of 5 × 10^5^ cells/ml. Post-recovery, the cells were labeled with dsFLUOR dye (Promega, E2671) for 2 min at a ratio of 1 μl dye per 4 ml of cell suspension. Preparing for FACS, the cells were treated with 10 μg/ml dihydroethidium (DHE), freshly made from a stock solution of 5 mg DHE in 500 μl dimethyl sulfoxide (DMSO), and incubated for 20 min at 37°C in the dark. Subsequently, the cells were washed three times with CMM containing 4% FCS (1200 × *g* for 5 min), which had been pre-filtered through a 50 μm filter to eliminate any FCS aggregates that might affect the FACS process. Lastly, the cells were resuspended at a concentration of about 2 × 10^7^ cells/ml for sorting. Sorting was carried out for five independent biological replicates each, uninduced or induced for RNAi for 5 days. For each replicate, ∼2 × 10^8^ cells were sorted in two equal-sized batches. To reduce nonspecific staining, sorting was conducted at a temperature of 10°C. Between 1.7 × 10^6^ and 3.9 × 10^6^ cells were recovered after sorting for cells with kDNA loss. Sorted cells were cultured for 80 h to produce sufficient material for PCR amplification of RNAi inserts; total genomic DNA was extracted from a total of 6.6 × 10^7^ to 1.5 × 10^8^ cells, depending on the replicate.

### DNA deep sequencing

To amplify RNAi target fragments from the genomic DNA, we followed a previously described PCR protocol using LIB2f and LIB2r primers, with 23 amplification cycles [39]. After amplification, DNA was sent for Illumina deep sequencing (BGI, Hongkong; 100PE, HiSeq 4000). Paired end sequences were scanned for the presence of each primer subsequence (F = CCCCTCGAGG, R = ATCAAGCTTGGCC) and trimmed using a custom script to remove extraneous bases as appropriate. The resulting fasta output files for F, R and UNK (unknown) for each sample were aligned to the *T. brucei* TREU927 genome (Release 42/v5.1 from TriTrypDB.org) using bowtie2 (version 2.2.7) [43] and parameters –very-sensitive -p 20 –no-unal. The resulting BAM files were sorted and indexed using SAMtools (version 1.12) [44], and alignment counts to predicted genes were generated from the BAM files with the appropriate bed file and BEDtools (v2.23.0) [45] using the multicov -bams *.bam command. The raw gene counts output from BEDtools was subsequently processed in R.

### RIT-seq data analysis

The statistical analysis of the RIT-seq data was performed in the R environment using edgeR package [46]. The number of reads mapping to each CDS for each condition (5 days induced sorted, 5 days noninduced sorted, and day 0 nonsorted populations; 5 replicates each) were normalized and log-transformed by the function calcNormFactors [trimmed mean of M-values (TMM) normalization].

### Construction of RNAi cell lines

For RNAi-mediated knockdown in bloodstream form parasites, the inducible stem-loop RNAi construct targeting gene *Tb927.8.4240* was generated using the pQuadra system [47]. Nucleotides 1–530 of the *8.4240* gene were amplified via PCR using primers 1722 and 1723 (Supplementary Table ST1). The oligonucleotides included specifically designed BstXI restriction sites to facilitate directional cloning [47]. Ligation with BstXI‐digested pQuadra1 and pQuadra3 plasmids generated pQuadra‐8.4240, containing inverted repeats of the PCR product separated by a spacer region, which were confirmed by sequencing the plasmid using primers 116, 102 and 82 (Supplementary Table ST1). The NotI-linearized construct was then introduced via nucleofection [48] into *T. brucei* Lister 427 ‘single marker’ bloodstream form cells, which express T7RNAP and TetR [49]. Transgenic cell lines harboring the inducible RNAi cassette were selected with phleomycin. Subsequently, kDNA-independent (γL262P) and kDNA-dependent control (γWT) cell lines were constructed in this RNAi background by transfection with linearized pEnT6-PURO-γL262P and -γWT plasmids, respectively, as described previously [49].

For knockdown in procyclic insect stage parasites, nucleotides 452–941 of the *Tb927.8.4240* gene were PCR-amplified with primers RNAi_fwd and RNAi_rev (Supplementary Table ST1) and cloned in stem-loop configuration into a modified version of pLew100 [49]. Procyclic cell line 29.13 [49], expressing T7RNAP and TetR, was grown in SDM-79 medium with 10% FCS, transfected with the NotI-linearized RNAi construct, and stable transgenic RNAi clones selected as described before [32].

### Quantitative real-time reverse transcription PCR

RNA from 1 × 10^8^ cells was isolated using the RNeasy Mini Kit (Qiagen). RNA quantity and quality were assessed by measuring A260/A280 and A260/A230 ratios using a Nano-Drop instrument (Thermo Scientific). RNA samples consistently showed ratios of ∼2.1 and 2.3, respectively, indicating high purity. Complementary DNA (cDNA) was synthesized from 1 μg of total RNA using the High-Capacity cDNA Reverse Transcription Kit (Applied Biosystems), following the manufacturer’s instructions. ‘Minus RT’ control reactions were prepared in parallel by replacing the reverse transcriptase with 1 μl nuclease free water. Each qPCR reaction was carried out in a final volume of 20 μl containing 10 μl SYBR Green Master Mix (Applied Biosystems), 2 μl cDNA template, or 2 μl of a 1:50 diluted cDNA for the reference gene reaction (see below), 2 μl each of forward and reverse primer (0.75 μM final concentration) and 4 μl nuclease free water. To measure *Tb927.8.4240* mRNA in samples from RNAi studies in bloodstream forms, we used primer pair 1777/1778 (see Supplementary Table ST1 for all quantitative real-time reverse transcription PCR primers) and a Roche LightCycler instrument, for RNAi studies in procyclic forms we used or primer pair 8.4240_qPCR_fwd/8.4240_qPCR_rev and a Bio-Rad C1000 Thermal Cycler. Cycling conditions consisted of an initial activation step at 95°C for 2 min, followed by 40 cycles of 95°C for 15 s, 60°C for 30 s, and 72°C for 30 s. Melt curve analysis was performed at the end of the amplification program to confirm assay specificity; all reactions produced a single peak consistent with amplification of a single product. No amplification was detected in ‘minus RT’ controls, confirming the absence of detectable genomic DNA contamination. Analysis of the data was performed using the 2^-ΔΔCT method for relative quantification [50] with telomerase reverse transcriptase (*Tb927.11.10190*; primers 1358/1359) and β-tubulin (e.g. *Tb927.1.2330*; primers b-tub_qPCR_fwd/b-tub_qPCR_rev) as the reference transcripts for bloodstream and procyclic form samples, respectively [51]. Each experiment was performed using three independent biological replicates, with three technical replicates per reaction.

### Construction of a cell line expressing tagged *Tb*927.8.4240

C-terminal *in situ* hemagglutinin (HA) tagging of the *Tb927.8.4240* gene was done using plasmids of the pMOtag series [52] as templates for PCR amplification with primers defining the sites of homologous recombination, followed by stable transfection of procyclic cell line 29.13 as described previously [32].

### Standard fluorescence microscopy

Standard fluorescence microscopy was performed as described previously [41]. Briefly, cells were fixed with 2% (w/v) cold formaldehyde in phosphate buffered saline (PBS), washed once with PBS (1000 × *g*, 5 min), and mounted on microscopy slides. DNA was visualized using Prolong Gold antifade reagent with 4′,6-diamidino-2-phenylindole (DAPI; Life Technologies), and images were captured using a Retiga 2000R Mono Cooled charged-coupled device camera attached to an Axioscope 2 or Axioimager Z2 (Carl Zeiss MicroImaging, Inc.) using either Plan-Apochromat 63× (1.40 NA) or Plan-Apochromat 100× (1.40 NA) phase-contrast objectives. The relative amount of DAPI-stained DNA was quantified using ImageJ software [53].

### Ultrastructure expansion microscopy

Coverslips (12 mm, Epredia) were functionalized with poly-D-lysine (A3890401, Gibco) at room temperature for 30 min, followed by three washes with deionized water. Cells were then spread onto the coverslips and allowed to settle at room temperature for 10 min, fixed with 4% paraformaldehyde for 10 min and permeabilized with 0.2% Triton X-100 in PBS for 5 min. Cells were washed twice with PBS and samples blocked with 4% bovine serum albumin (BSA) in PBS. Primary and secondary antibodies (see below for specifications) were diluted in PBS containing 2% BSA and were incubated for 45 min in between PBS washes. After the last wash with PBS, cells were anchored in PBS containing 0.7% formaldehyde (Sigma) and 1% acrylamide (AA; Sigma) at 37°C for 2 h. Gelation was performed using a freshly prepared solution composed of 19% sodium acrylate (Sigma), 10% AA, and 0.1% N,N'-methylenebisacrylamide (Sigma), supplemented with 0.5% ammonium persulfate (Thermo Fisher) and 0.5% tetramethylethylenediamine (Thermo Fisher). For gelation, 35 µl droplets of gelation solution were placed onto parafilm within a pre-cooled humidity chamber. Coverslips containing anchored cells were inverted onto droplets and incubated on ice for 5 min, followed by incubation at 37°C for 30 min to allow polymerization.

Gels were gently detached from the coverslips by incubating in 1 ml denaturation buffer (200 mM sodium dodecyl sulphate, 200 mM NaCl, 50 mM Tris in deionized water, pH 9) with gentle shaking at room temperature for 15 min. Subsequently, gels underwent denaturation in the same buffer at 95°C for 30 min. After denaturation, gels were washed three times for 5 min each with PBS, then incubated for 1 h at room temperature with gentle agitation in PBS containing DAPI (5 µg/ml; Sigma) for DNA staining. Following staining, gels were expanded overnight in deionized water.

Antibodies were used as follows:

- anti-HA tag antibody, rabbit, 1:1000 (Thermo Fisher)
- Goat anti-Rabbit IgG (H + L) Cross-Adsorbed Secondary Antibody, Alexa Fluor™ 594, 1:1000 (Thermo Fisher)
- Mouse monoclonal anti-TAC102 [54], 1:1000
- Goat anti-Mouse IgG (H + L) Cross-Adsorbed Secondary Antibody, Alexa Fluor™ 488 (Thermo Fisher)
- Guinea pig anti-α-tubulin, 1:500 (ABCD Antibodies)
- Guinea pig anti-β-tubulin, 1:500 (ABCD Antibodies)
- anti-guinea pig IgG Alexa Fluor 647, 1:500 (Sigma–Aldrich)

Expanded gels were sectioned and mounted onto poly-D-lysine-coated 25 mm glass-bottom dishes (Cellvis, 35 mm dish with 20 mm microwell #1.5 cover glass). Z-stack imaging was performed using a Nikon Ti2 Kinetix Widefield Microscope equipped with a 100× objective (NA = 1.45). Imaging parameters included a z-step size of 0.2 µm and a pixel size of 65 nm. Images were deconvolved using Huygens HRM software and analyzed in ImageJ [53].

## Results

### Development of a whole-genome scale RNAi screen for kDNA loss

We designed a screening approach based on a combination of genome-wide, inducible RNAi, a novel kDNA staining protocol, and FACS of kDNA° cells; an overview is presented in Supplementary Fig. S1. A key tool in our screening approach is the ability to genetically engineer bloodstream form *T. brucei* cells that are viable in the absence of kDNA [55]. This is achieved by expressing a mutated copy of the γ subunit (γL262P) of the mitochondrial F_1_F_O_-ATPase, allowing for the creation of a mitochondrial membrane potential in the absence of kDNA-encoded genes [55]. We transfected these kDNA-independent *T. brucei* cells with a whole-genome RNAi library [56, 57], achieving coverage of 60%–87% for the 11 megabase chromosomes and representation of 98.5%–99.1% of the 11 832 annotated transcribed genes, based on Illumina sequencing of RNAi inserts PCR-amplified from transfected cells and mapping of reads to the *T. brucei* TREU927 reference genome (Supplementary Table ST2). Another essential advancement was our development of an *in vivo* DNA staining protocol that allowed the sorting, by flow cytometry, of trypanosome cells that had lost their kDNA. Initial attempts using DHE, a dye previously reported to be specific for kDNA [58], did not yield a sufficient separation of kDNA^+^ and kDNA° cells by flow cytometry. However, subsequent trials of co-staining with DHE and dsFLUOR [59] resulted in a clear, if imperfect, separation of kDNA^+^ and kDNA^0^  *T. brucei* cells, as shown in Supplementary Fig. S1B.

As a potential limitation of our screen, we note that some previously identified kDNA maintenance factors may be specific for maxicircle replication, such as primase PRI1 [60]. Loss of maxicircles alone may not result in a sufficient decrease of kDNA staining for identification in the screen we developed.

### Identification of putative kDNA maintenance factors

In previous studies, RNAi-mediated ablation of kDNA maintenance factors in bloodstream form parasites has been shown to result in kDNA loss after 3–5 days [35, 61]. To maximize the sensitivity of our screen, we therefore induced RNAi in our library cells with tetracycline (tet) for 5 days. Another consideration for choosing this timeframe was to minimize interference by indirect effects: ablation of mitochondrial protein import, for example, would ultimately also result in kDNA loss due to secondary depletion of kDNA maintenance proteins. As kDNA-independent *T. brucei* still rely on other mitochondrial processes for survival, however [55], cells with such RNAi inserts will be depleted from the population within a few days.

After RNAi induction, kDNA° cells were isolated by FACS (Supplementary Fig. S1A). After extraction of total DNA from the kDNA° cells, RNAi inserts were amplified by PCR [39] and sequenced on an Illumina platform (100 bp paired-end) using an amplification-free library preparation protocol (sample ‘Day 5 + tet kDNA^0^’). For controls, we processed samples from (i) the full RNAi cell library prior to induction (‘Day 0′) and (ii) from un-induced cells grown in parallel to the induced culture for 5 days, and also sorted for kDNA loss (‘Day 5 −tet kDNA^0^’). The ‘Day 0′ control accounts for relative enrichment of RNAi library inserts in kDNA° cells after RNAi induction compared to the starting library. Comparison with the ‘Day 5 −tet kDNA^0^’ sample aimed to distinguish gene fragments specifically enriched due to RNAi induction from those lost due to background noise or nonspecific effects. Illumina reads were mapped to a *T. brucei* reference genome [62] and differentially abundant reads per gene between tested conditions were determined.

**Figure 1.**
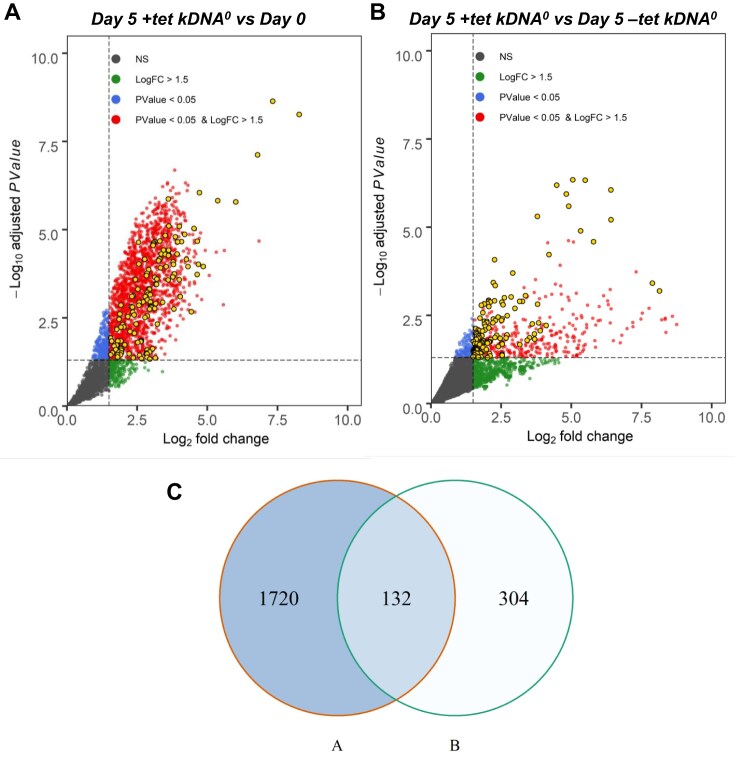
Identification of putative kDNA maintenance factors by genome wide RNAi screening. (**A, B**) Volcano plots showing the enrichment of genes in ‘Day 5 + tet kDNA^0^’ compared to ‘Day 0′ (**A**) and compared to ‘Day 5 −tet kDNA^0^’. (**B**) Each dot represents a gene. Significant enrichment is indicated for genes with a log_2_ fold change (log_2_FC) >1.5 and an adjusted *P*-value of <0.05, shown in red. Significantly enriched genes shared by both comparisons are shown as yellow dots. Blue, green, and gray dots represent genes with a log_2_FC <1.5, an adjusted *P*-value ≥ 0.05, or both (nonsignificant, NS), respectively. The vertical dashed line represents the log_2_FC threshold of 1.5, and the horizontal dashed line indicates the *P*-value threshold of 0.05. Five biological replicates were used for all samples (*n* = 5). (**C**) Venn diagram representing the intersection of genes identified in both comparisons.

Figure 1A and B show volcano plots, with thresholds for statistical significance set at log_2_ fold-change >1.5 and *P* <0.05. These criteria identified 1852 and 436 candidate genes, respectively, with an overlap of 132 genes between the two conditions (Fig. 1C, detailed in Supplementary Table ST3). While the precise number of genes required for kDNA maintenance is unknown, it is likely from the large number of hits identified under each condition that a considerable portion of the identified genes represent false positives. For example, only a small fraction of the genes from both hit lists encode proteins with experimentally determined mitochondrial localizations [36], contrary to what would be expected for most kDNA maintenance factors (Supplementary Table ST3). Specifically, the ‘Day 5 + tet kDNA^0^’ versus ‘Day 0′ and ‘Day 5 + tet kDNA^0^’ versus ‘Day 5 −tet kDNA^0^’ hit lists included only 249 (13.4%) and 85 (19.5%) genes encoding proteins with mitochondrial localization. Indeed, these percentages are similar to the percentage of genes encoding mitochondrial proteins (1650, or 14.0%, according to the latest estimate) [36] among all 11 832 annotated genes in the reference genome [62]. In addition, of the 63 genes reported to date [36] that encode kDNA-associated proteins, only 11 and 13, respectively, were included in our two hit lists (Supplementary Table ST3), suggesting false negatives as well. Notably, however, the 132 genes identified in both comparisons showed an enrichment for genes encoding mitochondrial proteins (32 genes, or 24%), including seven previously confirmed to be associated with the kinetoplast [36] (Fig. 1C and Supplementary Table ST3).

**Figure 2.**
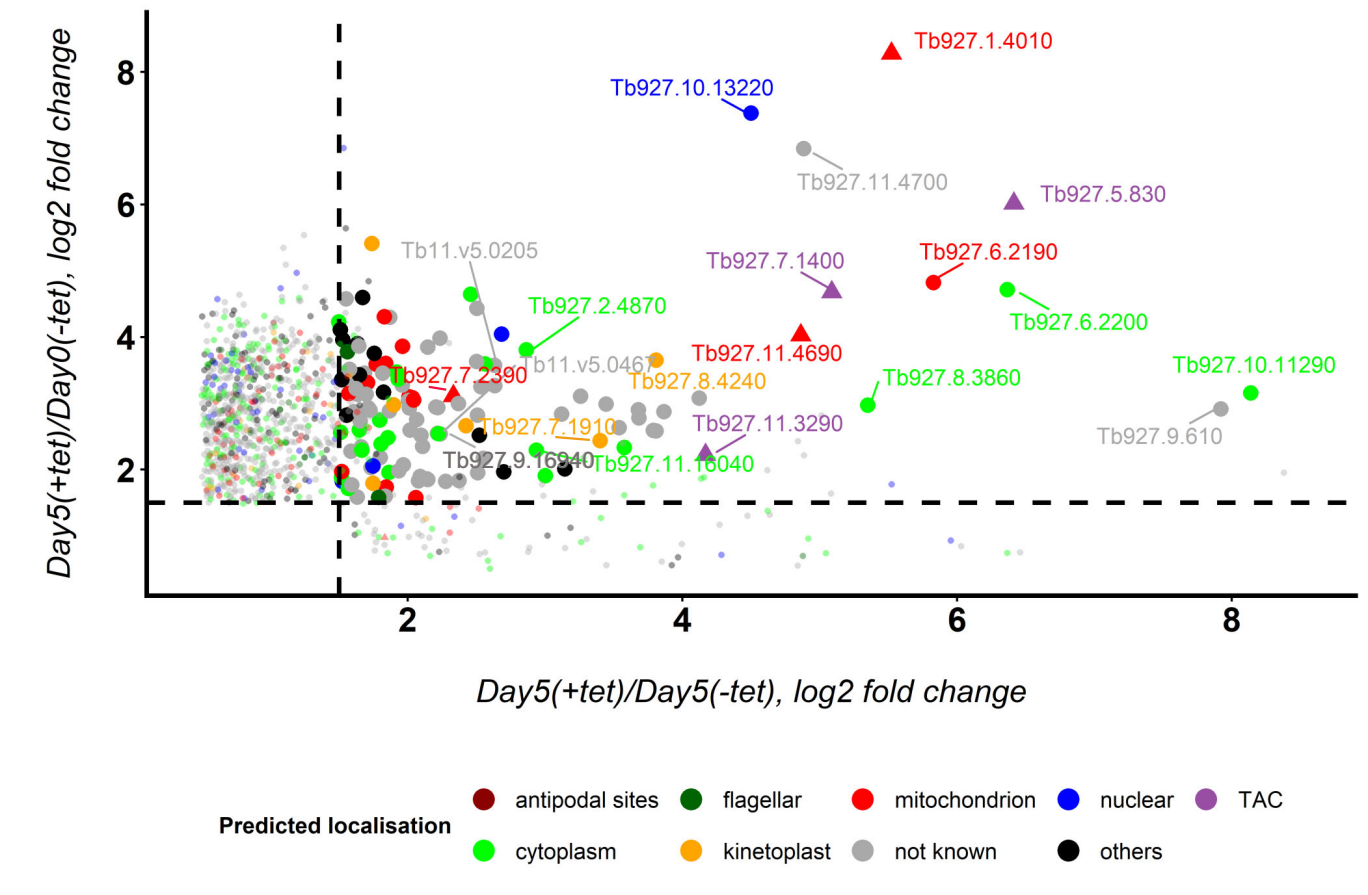
Visualization of primary set of 132 candidate genes implicated in kDNA maintenance, as identified through RNAi library screening. The visualized data include metadata on protein localization from TrypTag.org [36, 63, 64], integration with published mitoproteome analyses [65–67], and previously identified kDNA maintenance factors (triangles; ref [16] and references therein). Statistically significant changes (adjusted *P*-value < 0.05) are indicated by larger circles. For clarity and reference, TriTrypDB gene identifiers are indicated for the top 20 ranked candidates based on the false discovery rate (FDR) in the y-axis comparison, detailed in Table 1 and Supplementary Table ST3.

We therefore considered these 132 genes as our primary list of candidates. Figure 2 visualizes this selection, including published genome-wide data on localization [36, 63, 64], the experimentally determined mitochondrial proteome of *T. brucei* [65–67], as well as currently known kDNA maintenance factors [16] (see also Supplementary Table ST3).

Encouragingly, when candidates were ranked based on the FDR for the ‘Day 5 + tet kDNA^0^’ versus ‘Day 5 −tet kDNA^0^’ comparison, the top 20 included six genes encoding experimentally confirmed kDNA maintenance factors (four TAC components and two enzymes involved in replication): *TAC60* [68] (*Tb927.7.1400*, rank 1), *primase 2* [22] (*Tb927.1.4010*, rank 2), *TAC65* [69] (*Tb927.5.830*, rank 4), *mitochondrial DNA polymerase I protein B* [29] (*Tb927.11.4690*, rank 5), *p166* [33, 70] (*Tb927.11.3290*, rank 11) and *TAC102* [54] (*Tb927.7.2390*, rank 16) (Table 1 and Supplementary Table ST3).

**Table 1.** Top 20 candidates from the list of 132 primary candidates, ranked according to lowest FDR in the ‘Day 5 + tet kDNA^0^’ versus ‘Day 5 −tet kDNA^0^’ comparison (Supplementary Table ST3)

Rank	TriTrypDB ID	TriTrypDB annotation	kDNA loss confirmed after knockdown	kDNA association confirmed	Mitochondrial localization confirmed	Reference(s) (PMID)
1	*Tb927.7.1400*	Tripartite attachment complex protein 60	Yes	Yes	Yes	29287109
2	*Tb927.1.4010*	Primase 2	Yes	Yes	Yes	21257796
3	*Tb927.10.13220*	Cyclophilin type peptidyl-prolyl *cis-*trans isomerase, putative				23556014
4	*Tb927.5.830*	Tripartite attachment complex protein 65	Yes	Yes	Yes	27436903
5	*Tb927.11.4690*	Mitochondrial DNA polymerase I protein B	Yes	Yes	Yes	12150917
6	*Tb927.11.4700*	Prostaglandin f synthase				
7	*Tb927.8.4240*	Hypothetical protein, conserved		Yes	Yes	36804636, 32579605
8	*Tb927.6.2200*	DJ-1 family protein, putative			Yes	37669165
9	*Tb927.8.3860*	Cytosolic iron-sulfur protein assembly 1				30346997
10	*Tb927.6.2190*	Hypothetical protein, conserved			Yes	37669165, 28485388
11	*Tb927.11.3290*	Tripartite attachment complex protein 166 (p166)	Yes	Yes	Yes	18059470, 35709300
12	*Tb927.9.16940*	Variant surface glycoprotein (pseudogene), putative				
13	*Tb927.11.16040*	Prefoldin subunit, putative				
14	*Tb11.v5.0467*	Hypothetical protein, conserved				
15	*Tb927.9.610*	Hypothetical protein (pseudogene)				
16	*Tb927.7.2390*	Tripartite attachment complex protein 102	Yes	Yes	Yes	27168148
17	*Tb927.10.11290*	Hypothetical protein, conserved				
18	*Tb927.7.1910*	Pyridoxal phosphate containing glycine decarboxylase, putative			Yes	37669165
19	*Tb927.2.4870*	Kinetoplastid-specific dual specificity phosphatase, putative				
20	*Tb927.6.1290*	Hypothetical protein, conserved				

In addition to these six genes, four other candidates within the top 20 encode proteins with experimentally confirmed mitochondrial or kinetoplast locations (Table 1 and Supplementary Table ST3). *Tb927.8.4240* (rank 7) is annotated in TriTrypDB as a hypothetical gene, universally conserved among the sequenced kinetoplastids. A C-terminally tagged version of the 184 kDa protein has been localized to the kinetoplast [63]. Notably, *Tb927.8.4240* is a paralog of the adjacent gene *Tb927.8.4230*, previously identified as encoding a putative interactor of LigK-β in a proximity labelling approach [71]. LigK-β and LigK-α are kDNA-associated DNA ligases [26]; only LigK-α expression has been successfully knocked down and shown to be essential for kDNA replication [26]. While *Tb*927.8.4230 localizes exclusively at the APS, analogous to *T. brucei* LigK-β [26, 71], *Tb*927.8.4240 is reportedly localized in the proximity of kDNA, but not at the APS themselves [71].

*Tb927.6.2200* (rank 8) and *Tb927.6.2190* (rank 10) are neighboring genes, and close inspection of the mapped RNAi fragments revealed a fragment that included parts of both genes. It is therefore possible that only one of these two genes is a genuine hit, while the other is a ‘collateral’ hit. *Tb*927.6.2200 was predicted to be a mitochondrial protein based on its significantly reduced abundance in a mitochondrial fraction after ablation of ATOM40, a protein critical for mitochondrial protein import [67]. The human homolog of this protein, DJ-1 (encoded by *Park7*), is also mitochondrial and is involved in protection from oxidative stress [72]. *Tb*927.6.2190 is another ‘conserved hypothetical’ protein; its mitochondrial location was experimentally confirmed in multiple studies, and its expression appears to be regulated during the cell cycle [73]. The final gene of interest in this subset, *Tb927.7.1910* (rank 18), encodes a component of the glycine cleavage complex [74], a crucial element in mitochondrial metabolism and function. This finding highlights the complex’s possible involvement in kDNA maintenance, potentially expanding the list of metabolic enzymes with such a dual function [75].

Six of the top 20 hits do not appear to encode mitochondrial proteins. *Tb927.10.13220* (rank 3) is annotated as a cyclophilin-type isomerase. Although some members of this family, like Cyclophilin D, have important mitochondrial functions [76], this gene has been predicted to encode a member of the Fam66 family of cell surface proteins [77]. *Tb927.8.3860* (rank 9) has been experimentally confirmed as a component of the cytosolic iron-sulfur cluster assembly complex [78], which makes a putative association with kDNA maintenance unexpected. *Tb927.11.16040* (rank 13) is conserved among kinetoplastids, shows some homology to prefoldin-like chaperones and appears to be located in the cytoplasm. *Tb11.v5.0467* (rank 14), *Tb927.10.11290* (rank 17), and *Tb11.v5.0205* (rank 20), despite their conservation among kinetoplastids, possess uncertain cellular localizations [63], and lack identifiable functional motifs, which adds a layer of complexity to the functional characterization of these genes. Gene *Tb11.v5.0205* is identical in sequence to *Tb927.10.6130*. A tagged version of the encoded protein has been localized to the cytoplasm [63]. The *Tb11.v5.0467* entry in TriTrypDB includes a 5′ UTR of nearly 3 kb that, in part, shares 100% identity with much of the *TAC102* coding sequence, suggesting that the contig that contains *Tb11.v5.0467* might be an assembly artefact and that this gene is a false positive hit.

Finally, the top 20 candidates include a further three genes that are probably false positive hits. *Tb927.916940* (rank 12) and *Tb927.9.610* (rank 15) are annotated as pseudogenes. Tb927.11.4700 (rank 6) is likely a ‘collateral hit’: it is located next to *Tb927.11.4690* (rank 5, see above) in the genome, and, similar to *Tb927.6.2200* and *Tb927.6.2190*, a close inspection of the mapped RNAi fragments revealed a fragment that included parts of both genes. Thus, the identification of *Tb927.11.4700* may result from its genomic proximity to a true positive hit rather than involvement in kDNA maintenance.

### Confirmation of *Tb*927.8.4240 as a novel protein involved in kDNA maintenance

Hypothetical protein *Tb*927.8.4240 (rank 7) was our top-ranked hit with evidence for mitochondrial localization that had not been previously characterized. We therefore prioritized this hit for follow-up studies. The 184 kDa protein product has no recognizable features or domains, except an intrinsically disordered region at its C-terminus (residues 1464–1689) predicted by InterPro [79]. The structural model generated by AlphaFold (https://alphafold.ebi.ac.uk/entry/Q57W11) [80] has only a few domains of high confidence, with only limited structural similarity to any other proteins in structural databases, according to Foldseek [81]. Interestingly, a Foldseek search with the *Tb*927.8.4240 structure predicted by AlphaFold 3 [82] identifies low but significant similarity of the central region (around residues 575–1000) to various ATPase domain proteins, including chromosomal replication initiator protein DnaA from bacteria.

As reported previously, *Tb927.8.4240* and its genomic neighbor in *T. brucei, Tb927.8.4230* (a gene encoding a smaller protein of 119 kDa) are the closest homologs of each other in the *T. brucei* genome (E-value 9e-41 in a protein BLAST) [71]; they are likely paralogs that resulted from a gene duplication event. A search for potential trypanosomatid orthologs and their genomic locations using the Tri-Tryp database (www.tritrypdb.org) [83] confirms that both genes are highly conserved and usually syntenic in trypanosomatids. An exception is *Trypanosoma cruzi*, where a gene specific for that species has been inserted between the two paralogs. Interestingly, in *Trypanosoma congolense* IL3000, both genes—although still syntenic with their orthologs in other trypanosomatids, appear to have degraded and are annotated as pseudogenes *TcIL3000.A.H_000 602 900* and *TcIL3000.A.H_000 603 000* on chromosome 8. Orthologs of *Tb927.8.4240* and *Tb927.8.4230* appear to be absent from the free-living genus *Bodo* [71], the closest known relative of trypanosomatids. A syntenic *Tb927.8.4240* ortholog is present in the early-branching trypanosomatid *Paratrypanosoma confusum*, but a *Tb927.8.4230* ortholog is missing in this species. This suggests a scenario where the gene duplication that gave rise to the two paralogs occurred after *P. confusum* had branched off; however, it is also possible that the *Tb927.8.4230* ortholog was secondarily lost in that species. Supplementary Fig. S2 shows sequence conservation for the orthologs from five selected trypanosomatids, *T. brucei, Trypanosoma vivax, T. cruzi, Leishmania major*, and *Crithidia fasciculata*. Overall, within the *Trypanosoma* genus, *Tb*927.8.4240 and *Tb*927.8.4230 protein sequences are 57%–59% and 43%–47% identical, respectively (Supplementary Fig. S2A). Areas of relatively high conservation are concentrated in six and two clusters, respectively (Supplementary Fig. S2B and C).

**Figure 3.**
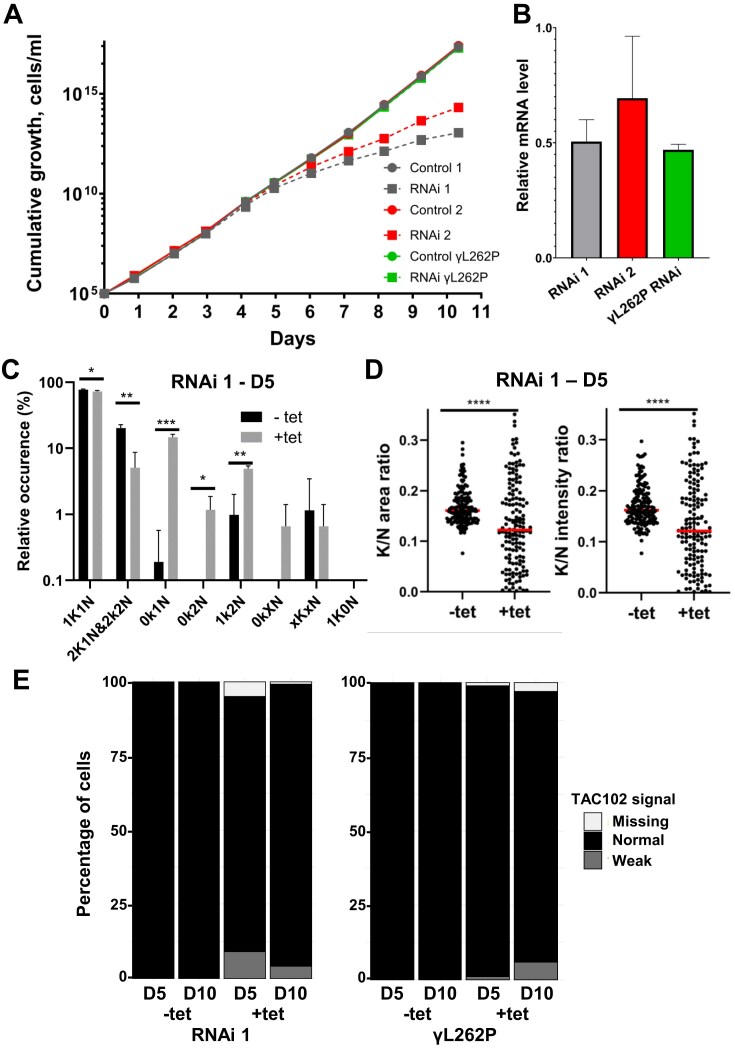
Phenotypic analysis of *Tb*927.8.4240 ablation in bloodstream form *T. brucei*. (**A**) Growth phenotype upon *Tb*927.8.4240 RNAi knockdown in a wild-type background (two independent RNAi clones; gray and red) is rescued in a kDNA-independent background (introduction of γL262P mutation; green). (**B**) Levels of mRNA measured by quantitative RT-PCR, at day 3 post induction (*n* = 3). (**C**) Quantification of relative occurrence of kDNA (K) and nuclei (N) in DAPI stained cells from induced (+tet) and noninduced (−tet) RNAi cell line 1 populations at day 5 post induction (*n* > 100 cells for each triplicate; unpaired *t*-test *P* <0.05*, *P* <0.01**, *P* <0.001***). (**D**) The relative amount of kDNA to nucleus area (left panel) or fluorescence intensity (right panel) in 1K1N RNAi cell line 1 at 5 days post-induction (Mann–Whitney test; *P* <0.0001^****^). (**E**) *Tb*927.8.4240 RNAi knockdown in a wild-type or γL262P background has only a minor effect on TAC102, as assessed with a TAC-specific antibody and epifluorescence microscopy (see Fig 4A; *n* > 100 cells for each sample; paired *t*-test *P* <0.05).

To confirm the role of *Tb*927.8.4240 in kDNA maintenance, we ablated its expression in bloodstream form *T. brucei* by inducible RNAi (Fig. 3). In two independent RNAi clones, after 3 days of induction, mRNA levels were reduced to ∼50% and ∼70%, respectively (Fig. 3B), which resulted in a marked reduction in the population’s growth rate ∼5 days post-induction (Fig. 3A, gray and red dashed lines). Induction of RNAi in a kDNA-independent cell line (i.e. expressing γL262P) did not result in a growth phenotype (Fig. 3A, green lines), despite a comparable reduction in mRNA levels (Fig. 3B), confirming a kDNA-specific growth effect. After 5 days of RNAi induction, the percentage of cells in the population that had completely lost their kDNA had increased substantially: from ∼0.2% to > 10% for 0K1N cells (i.e. cells with 1 nucleus but no kDNA) and from 0% to ∼1% for 0K2N cells (Fig. 3C). In contrast, cells with replicated and segregated kDNA (2K1N and 2K2N) had decreased substantially. For cells that still possessed visible kDNA 5 days post-induction, a quantification of the kDNA-to-nucleus size ratio at the same time point revealed an overall reduction in average kDNA size and an increased variability in kDNA size (Fig. 3D). Even in the kDNA-independent RNAi cell line, which could be observed for a longer period after induction, the percentage of 0K1N cells remained relatively stable at 10% (Supplementary Fig. S3). Depletion of *Tb*927.8.4240 in WT cells or in γL262P cells by RNAi had only a very minor effect on the amount or localization of the kDNA-proximal TAC component TAC102 [15, 54] (Fig. 3E), ruling out a role for *Tb*927.8.4240 in TAC assembly or structure.

We also ablated *Tb*927.8.4240 expression in the procyclic insect stage of *T. brucei*, which resulted in phenotypes similar to those observed in bloodstream form cells (Supplementary Fig. S4). Reduction of the mRNA level to ∼30% (measured 2 days after RNAi induction) resulted in growth retardation from day 6 after RNAi induction (Supplementary Fig. S4A and B) and a significant reduction in the amount of kDNA per cell (Supplementary Fig. S4C). The relative proportion of 0K1N and 0K2N cells in the population appeared to be increased on day 5 post RNAi induction; however, this was not statistically significant (Supplementary Fig. S4D).

Overall, these results suggest an important and specific role of *Tb*927.8.4240 in kDNA maintenance. The incomplete cessation of cell growth in kDNA-dependent cells and the incomplete kDNA loss in both kDNA-dependent and -independent cells could suggest some intrinsic variability in parasite dependence on *Tb*927.8.4240 function, or it could be a consequence of variable degrees of RNAi efficiency at the single cell level—the measured reduction in mRNA levels to 50%–70% in bloodstream forms and to ∼30% in procyclic forms represent averages across the population. The somewhat milder phenotypes observed in procyclic compared to bloodstream cells after RNAi knockdown, despite a more efficient reduction in the level of mRNA, could indicate that procyclic cells require relatively less *Tb*927.8.4240 protein for kDNA maintenance or that the *Tb927.8.4240* mRNA is more efficiently translated in that lifecycle stage.

### *Tb*927.8.4240 shows a dynamic localization at and near the kDNA APS and at the kDNA disk periphery

**Figure 4.**
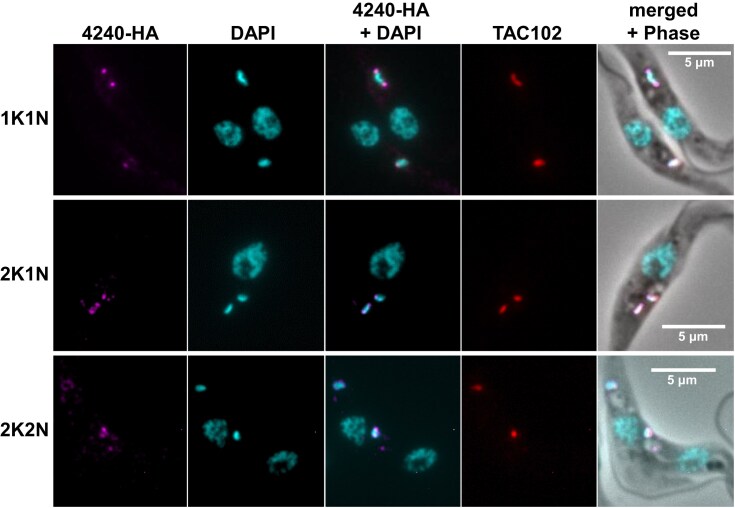
*Tb*927.8.4240 localization in procyclic *T. brucei* throughout the cell cycle assessed using standard epifluorescence microscopy. From left to right: *Tb*927.8.4240-HA tagged signal (magenta, anti-HA); nuclear and kDNA (cyan, DAPI); merge of anti-HA and DAPI; TAC102 (red, anti-TAC102); merge of anti-HA and DAPI with phase contrast image. Scale bar 5 μm.

To confirm the association of the *Tb*927.8.4240 protein with kDNA, we expressed a HA-tagged version in procyclic form *T. brucei* and first determined the approximate location by fluorescence microscopy (Fig. 4). Co-staining for DNA and for TAC102 suggested that the *Tb*927.8.4240 protein is located at the APS.

**Figure 5.**
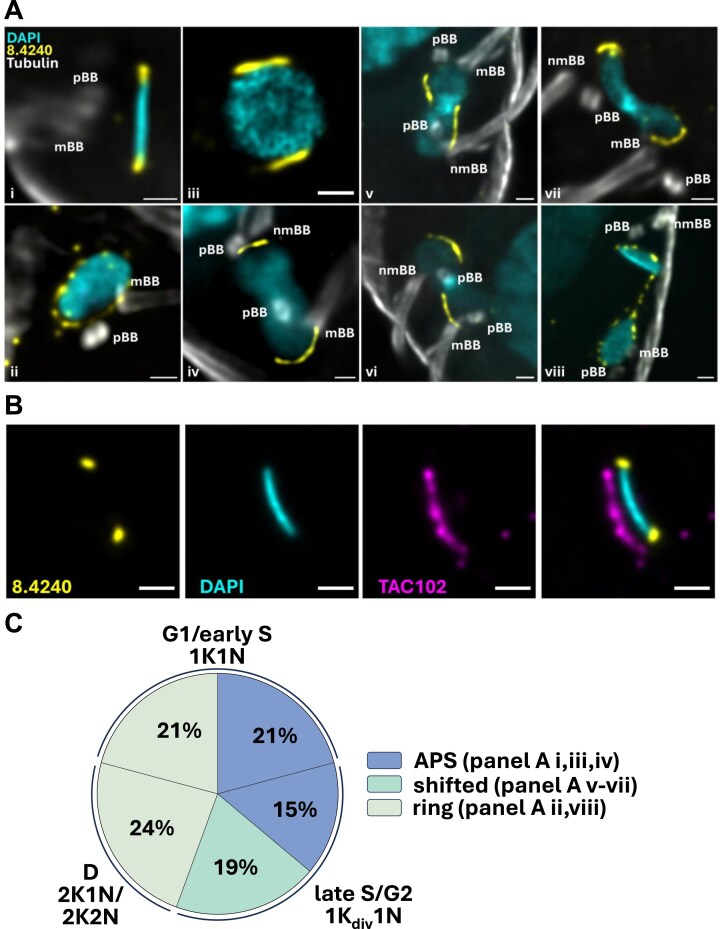
*Tb*927.8.4240 localization in procyclic *T. brucei* throughout the cell cycle, determined with U-ExM. (**A**) *Tb*927.8.4240 localization pre replication (images i–iii), in replicating networks (images iv–vii), and in segregating networks (image viii), from both lateral (image i) and axial views (images ii–viii). *Tb*927.8.4240 is shown in yellow, kDNA in cyan, tubulin in gray; pBB, pro-basal body; mBB, mature basal body, nmBB, new mature basal body. Scale bar: 1 µm. (**B**) U-ExM image of *Tb*927.8.4240 (yellow) co-stained with TAC102 (magenta); kDNA is shown in cyan. Scale bar: 1 µm. (**C**) Quantitation of *Tb*927.8.4240 localization during the kDNA cell cycle stages, as defined by Benz *et al*. [85] (G1, gap 1; S, synthesis; G2, gap 2; D, division), and as determined by U-ExM. Configuration 1K_div_1N represents cells with replicating kDNA, as in images iv–vii in panel (A), and as determined by kDNA shape and basal body configuration [86]: kDNA starts replicating when the new basal body pair appears and continues to replicate till the basal bodies separate after rotation. A total of 144 cells were analyzed.

To precisely localize *Tb*927.8.4240, we performed ultrastructure expansion microscopy (U-ExM; Fig. 5). Throughout the cell cycle, *Tb*927.8.4240 displayed distinct localization patterns when viewed from lateral and axial perspectives (Fig. 5A). In G1/early S-phase cells (1K1N), we observed two punctate signals consistent with APS localization and clearly distinct from the TAC when viewed laterally (Fig. 5Ai and B), while, when viewed axially, these signals either appeared at a punctate distribution around the edge of the disk (‘ring’, Fig. 5Aii) or in elongated shapes at opposite sites, presumably again reflecting APS localization (Fig. 5Aiii). These two distinct distributions were of similar frequency (Fig. 5C). In cells with dumbbell-shaped kDNA, i.e. in an advanced stage of replication, *Tb*927.8.4240 signals either exhibited similar elongated shapes with increased separation (Fig. 5Aiv) or seemed shifted to oppose each other along the replicating kDNA rather than remaining antipodal (Fig. 5Av–vii). The latter distribution was observed slightly more frequently (Fig. 5C). In segregating networks, the protein localization appeared as punctate rings again, with an additional localization along the ‘nabelschnur’ structure [84] between the disks (Fig. 5Aviii).

In summary, we found that the *Tb*927.8.4240 protein shows a dynamic, cell cycle-dependent localization.

## Discussion

Biochemical and imaging approaches have resulted in substantial progress in the identification of genes involved in replication and segregation of the mitochondrial genome of trypanosomatids [16, 36, 87]. Using a newly developed, genome-wide genetic screening approach, we have identified novel candidates for genes that are important in this process. We selected the gene with systematic ID *Tb927.8.4240*, annotated as a conserved hypothetical gene (www.TriTrypDB.org;) [83], for experimental follow-up.

Our bioinformatic analysis failed to identify any putative domains or motifs in the 184-kDa *Tb*927.8.4240 protein, except an intrinsically disordered region at its C-terminus. Similarly, the structure predicted by AlphaFold 2 has only few domains of high confidence and very limited similarity to any domains with characterized function [80]. Interestingly, however, the central region of the structure predicted by AlphaFold 3 shows some similarity to ATPase-domain containing proteins, including chromosomal replication initiator protein DnaA from bacteria.

Our gene expression knockdown by RNAi in bloodstream form *T. brucei* reduced the mRNA level to 50%–70% and produced a slow growth phenotype after 5 days, a timescale that is consistent with growth phenotypes obtained in bloodstream forms for other kDNA maintenance factors [21, 88, 89]. At day 5 post-induction, about 10% of cells had completely lost their kDNA (0K1N, or kDNA^0^). A substantial reduction in kDNA size was observed for most remaining cells, although an increase in kDNA size was observed for some cells. Parasites then continued to grow at a slower rate, with no further increase in kDNA° cells. It is uncertain why the percentage of kDNA° cells did not eventually reach 100%, even in a kDNA-independent genetic background, but relatively mild growth and kDNA-loss phenotypes are not unusual after RNAi-mediated ablation in bloodstream form *T. brucei*, even for essential kDNA maintenance genes ([89] and references therein). The most straightforward explanations are the incomplete ablation of *Tb927.8.4240* mRNA (see above) and the known variability of RNAi efficiency within cell populations [90].

**Figure 6.**
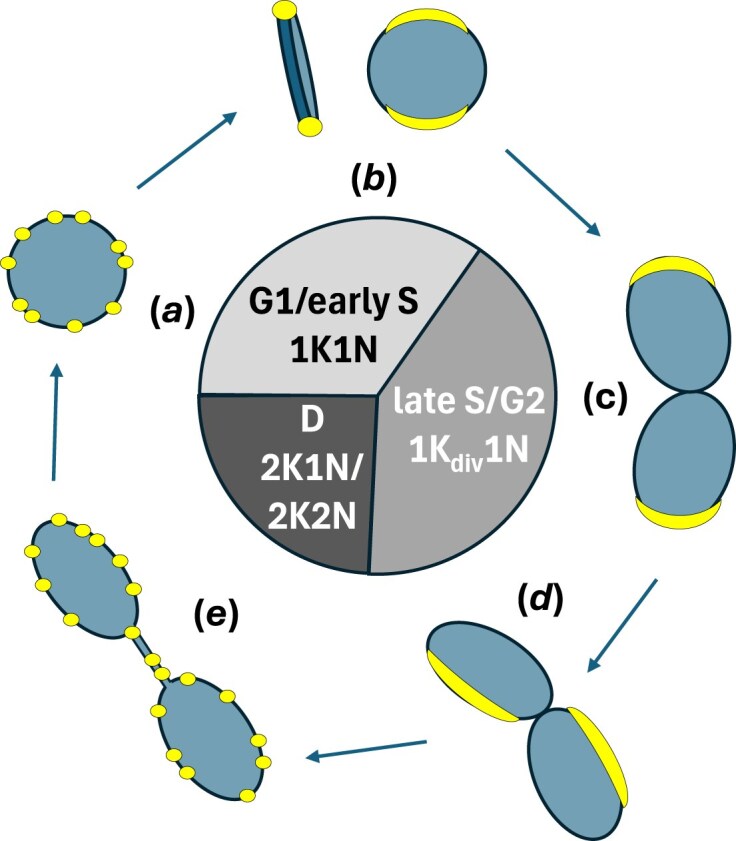
Model for dynamic localization of *Tb*927.8.4240 during kDNA replication. The model is based on the U-ExM studies shown in Fig. 5 and on the revised kinetoplast cell cycle stages proposed by Benz and colleagues [85] (abbreviations as in Fig. 5). Blue, kDNA; yellow, *Tb*927.8.4240 protein. Note that the respective size of the cell cycle stages in the diagram is drawn simply for convenience and not proportional to their actual duration. The three distinct states of localization are ring-like (a, e), antipodal (b, c) and shifted (d), in accordance with Fig. 5.

A V5-tagged version of the *Tb*927.8.4240 protein was previously reported to localize near the kDNA APS, but potential changes in localization during the cell cycle were not explored [71]. Our localization studies using HA-tagged protein and standard fluorescence microscopy as well as U-ExM showed a dynamic, cell cycle-dependent location of the *Tb*927.8.4240 protein. For cells at the beginning of kDNA replication, we observed two alternative localizations within the population: either punctate, distributed around the rim of the kDNA disk, resulting in a ring-like appearance (Fig. 6, state *a*), or signals consistent with localization at APS (Fig. 6, state *b*). Viewed axially, APS localization was not dot-like, as in images obtained by conventional microscopy [63], but extended along the edge of the kDNA (Fig. 6, state *a*), similar to the recently discovered replication factor MiRF172 [21]. In dumbbell-shaped (or bilobed) kDNA, characteristic of cells at an advanced stage of replication, the *Tb*927.8.4240 protein exhibited a similar localization pattern in ∼45% of cells (Fig. 6, state *c*), or its location was shifted along the edge of the elongated kDNA rather than remaining antipodal (Fig. 6, state *d*). It seems reasonable to assume that state *d* develops via state *c*, rather than directly from state *b*, as depicted in Fig. 6, although further experiments will be required to confirm this. This repositioning away from the APS is reminiscent of patterns observed after pulse labelling of replicating *T. brucei* minicircles with bromodeoxyuridine (BrdU), which led to the development of a model of *T. brucei* kDNA replication where back-and-forth oscillation of the kDNA disk facilitates a more equal distribution of daughter minicircles in the replicating network [91]. Later metabolic labelling experiments with the thymidine analogue 5-ethynyl-2′-deoxyuridine did not reproduce these patterns [92], and it was speculated that the DNA denaturation step required for BrdU antibody detection might have resulted in imaging artefacts, questioning the oscillation model [2].

Interestingly, in segregating networks we also observed association of some *Tb*927.8.4240 protein with the ‘nabelschnur’ (German for umbilical cord) in addition to redistribution to the punctate pattern around the rim of the disk seen in state *a* (Fig. 6, state *e*). As the nabelschnur represents maxicircle DNA that is stretched out between the segregating networks [84, 86], this observation suggests that *Tb*927.8.4240 is not exclusively involved in minicircle replication. Together with the increase in kDNA size that was observed for some bloodstream form cells after *Tb*927.8.4240 knockdown, this localization pattern might indicate a role in segregation as well. Previously, only two proteins have been reported that show association with the nabelschnur structure, the leucyl aminopeptidase *Tb*LAP1 [93] and *Tb*NAB70 [94]. Similar to *Tb*927.8.4240, both proteins show co-localization with the kDNA disk during replication (specifically with the kDNA periphery in the case of *Tb*NAB70) and, additionally, with the nabelschnur during segregation. Phenotypes for ablation are clearly distinct from what we observed for *Tb*927.8.4240, though: ablation of TbLAP1 results primarily in accumulation of 2K2N cells [93], while ablation of TbNAB70 prevents kDNA segregation and results in accumulation of 1K2N cells [94].

There is additional precedence for changes in localization of kDNA replication factors during the cell cycle. For example, DNA polymerases IC and ID only accumulate at APS in actively replicating networks, but are dissipated throughout the mitochondrial matrix during other phases of the cell cycle [95, 96]. Particularly relevant to our observation is the dynamic change in expression and localization of DNA LigK-α during the cell cycle in *C. fasciculata*, this enzyme being primarily observed on the faces of the kDNA disk in dividing cells, and having greater localization at the KFZ face in some cells and on the opposite face in others [97]. *Trypanosoma brucei* LigK-α is encoded by *Tb927.7.610*, the paralog of its neighboring gene, *LigK-β* (*Tb927.7.600*). According to proximity labelling studies, the LigK-β protein interacts with the *Tb*927.8.4240 paralog *Tb*927.8.4230 [71], and both proteins are localized at the APS [26, 71]. It is therefore tempting to speculate that *Tb*927.8.4240 dynamically interacts with DNA LigK-α, presumably specifically in dividing cells. The presence of a predicted intrinsically disordered region in *Tb*927.8.4240 is consistent with this idea, as the conformational malleability of these domains is well suited to facilitate dynamic interactions [98]. Co-folding of the *Tb*927.8.4240/*Tb*LigK-α and *Tb*927.8.4230/TbLigK-β pairs in AlphaFold 3 does not support direct interaction, however (ipTM scores of 0.22 and 0.56, respectively), suggesting any interaction might be indirect. This hypothesis needs to be tested further in future experiments. Orthologs for both DNA ligases are also neighboring genes in the *Bodo saltans* genome (BSAL_42 315 and BSAL_42 320, www.TriTrypDB.org;) [83]. Bodonids are free-living kinetoplastids that are a sister group of the trypanosomatids, have free minicircles and lack a kDNA network structure [99, 100]. It has been suggested that the network structure evolved in trypanosomatids to minimize minicircle loss during cell division [101]. Our search of the TriTrypDB database for orthologs of *Tb927.8.4230* and *Tb927.8.4240* did not find orthologs for either gene in *B. saltans*. However, we identified a syntenic ortholog for *Tb927.8.4240*, but not *Tb927.8.4230*, in the early-branching trypanosomatid *P. confusum*. This suggests a scenario where recruitment of *Tb*927.8.4240 for kDNA replication and subsequent gene duplication and specialization of paralog function evolved with the need for more sophisticated kDNA replication and segregation during trypanosomatid evolution. Future studies should systematically investigate the presence and absence of known kDNA replication and segregation factors in the rapidly growing number of kinetoplastid genomes [102].

Above we described the validation and characterization of *Tb*927.8.4240 as a novel kDNA maintenance factor. This, along with the identification of six known kDNA maintenance factors in our top 20 list (Table 1) is proof-of-concept that, in principle, the genetic screen that we designed for identification of kDNA maintenance factors is effective. However, the following findings suggest that there is room for improvement.

- Of the most recent list of 63 known genes encoding kinetoplast-associated proteins, which include dozens of genes where ablation has been reported to result in disruption of kDNA biogenesis [16, 36], only seven are present in our list of 132 candidate genes (Supplementary Table ST3 and Fig. 1). Thus, our screen produced a considerable number of false negatives.
- The vast majority of proteins that are important for kDNA maintenance are expected to have mitochondrial localization. Although there is precedence for proteins that are involved in kDNA maintenance and that have an additional role outside of the mitochondrion (e.g. mitochondrial topoisomerase 2, *Tb*927.9.5590 [55, 103]), and indeed we would expect some nonmitochondrial proteins with important roles in kDNA maintenance (e.g. for coordination of kDNA replication and segregation with the cell cycle), the number of proteins where this is the case is expected to be small. The 132 candidate genes include 32 genes (24%) where the protein product has been mapped to the mitochondrion [36]. Although this is a clear enrichment over the estimated 14% of all genes that, according to a recent estimate [36], encode mitochondrial proteins, a percentage of 76% nonmitochondrial proteins suggests a considerable number of false positives in our list. Indeed, the list includes many genes with known functions that are not related to kDNA biogenesis. Thus, our screen clearly produced a considerable number of false positive hits.
- As another potential limitation of our screen, we note that some previously identified kDNA maintenance factors may be specific for maxicircle replication, such as helicase PIF2 [23]. Loss of maxicircles alone might not result in a sufficient decrease of kDNA staining for isolation of cells in our FACS protocol. Similarly, negative regulators of kDNA replication such as the HslVU complex, where ablation results in an increase in the amount of kDNA [104], would not be identified in our screen.

How could the performance of the screen be improved to reduce the number of false negatives and false positives? One potential source of false positives could be secondary kDNA loss due to impaired mitochondrial function. Known components of the mitochondrial protein import machinery were absent from our list of 132 candidate genes, suggesting that our strategy to select against such indirect kDNA loss was at least partially successful. Nonetheless, future screens could include staining for an intact mitochondrial membrane potential as an indicator of healthy mitochondria. Another key step in our experimental design is separation of kDNA^+^ and kDNA° cells by FACS (Supplementary Fig. S1). Although this approach separated these cell types well, it was not perfect: in a control experiment with a pure population of kDNA^+^ cells, ∼0.5% of cells ended up in the kDNA^0^ gate (Supplementary Fig. S1B). To produce enough genomic DNA for efficient amplification of RNAi inserts by PCR, we cultured cells for 80 h after sorting (see the ‘Materials and methods’ section). Cells expressing γL262P tolerate kDNA loss well [55], but we noticed that in mixed populations of kDNA^+^ and kDNA° cells, the percentage of the former slowly increases. Therefore, it would be beneficial to further optimize the separation of kDNA^+^ and kDNA° cells by FACS and, also, the PCR protocol to allow efficient amplification of RNAi inserts directly after sorting. Finally, increasing the number of biological and technical replicates (we had used five biological replicates for the entire procedure from RNAi induction over sorting to library preparation and sequencing) would improve the identification of false hits with statistical methods.

Nonetheless, in addition to the characterized hit *Tb*927.8.4240, our hit list includes promising candidates for prioritization in future studies. This includes three candidates in our top 20 list with confirmed mitochondrial localization of the encoded proteins (*Tb927.6.2200, Tb927.6.2190, Tb927.7.1910*; Table 1) and 22 candidates further down in the list that also encode mitochondrial proteins (Supplementary Table ST3). Of particular note is *Tb927.5.520*, a gene encoding a putative stomatin-like protein, as proteins with the stomatin/prohibitin/flotillin/HflK/C domain have been reported as parts of complexes involved in maintenance of mitochondrial DNA [105].

In conclusion, we present an experimental strategy for a genetic screen that (i) has identified a novel kDNA maintenance factor, *Tb*927.8.4240, (ii) suggests further promising candidates for genes with important roles in kDNA maintenance, and (iii) points the way for second-generation screens with improved performance. Our characterization of *Tb*927.8.4240 suggests that the oscillation model for kDNA replication in *T. brucei* should be revisited, and that detailed and systematic phylogenetic studies of the evolution of known kDNA maintenance factors in kinetoplastids will be a feasible and fruitful endeavor.

## Supplementary Material

gkag493_Supplemental_Files

## Data Availability

Raw sequencing reads have been deposited at the NIH SRA under BioProject ID PRJNA1402727.

## Notes

Present address: Ophiomics, 1600-514 Lisbon, Portugal

Present address: Artidis, 4057 Basel, Switzerland

Present address: Institut Pasteur, Université Paris Cite, INSERM U1347, Paris, France
