# Supplementary material for: A genome-wide genetic screen identifies a novel kDNA replication protein in trypanosomes
Source: Nucleic Acids Res. 2026 May 21;54(10):gkag493. doi: 10.1093/nar/gkag493 (PMC13191291; doi:10.1093/nar/gkag493)
Supplement: gkag493_Supplemental_Files [file gkag493_supplemental_files.zip › Supplementary Figures.pdf]

A Genome-Wide Genetic Screen Identifies a Novel kDNA Replication Protein in Trypanosomes

Migla Miskinyte[1], Clirim Jetishi[2], Ana Kalichava[2], Alasdair Ivens[1], Martin Waterfall[1], Matt Gould[1], Lucy Glover[3], David Horn[3], Torsten Ochsenreiter[2], Achim Schnaufer[1,*]

[1]Institute of Immunology and Infection Research, University of Edinburgh, Edinburgh, EH9 3FL, UK

[2]Institute of Cell Biology, Faculty of Science, University of Bern, 3012 Bern, Switzerland

[3]Faculty of Life Sciences, University of Dundee, Dundee, DD1 5EH, UK.

Supplementary Figures

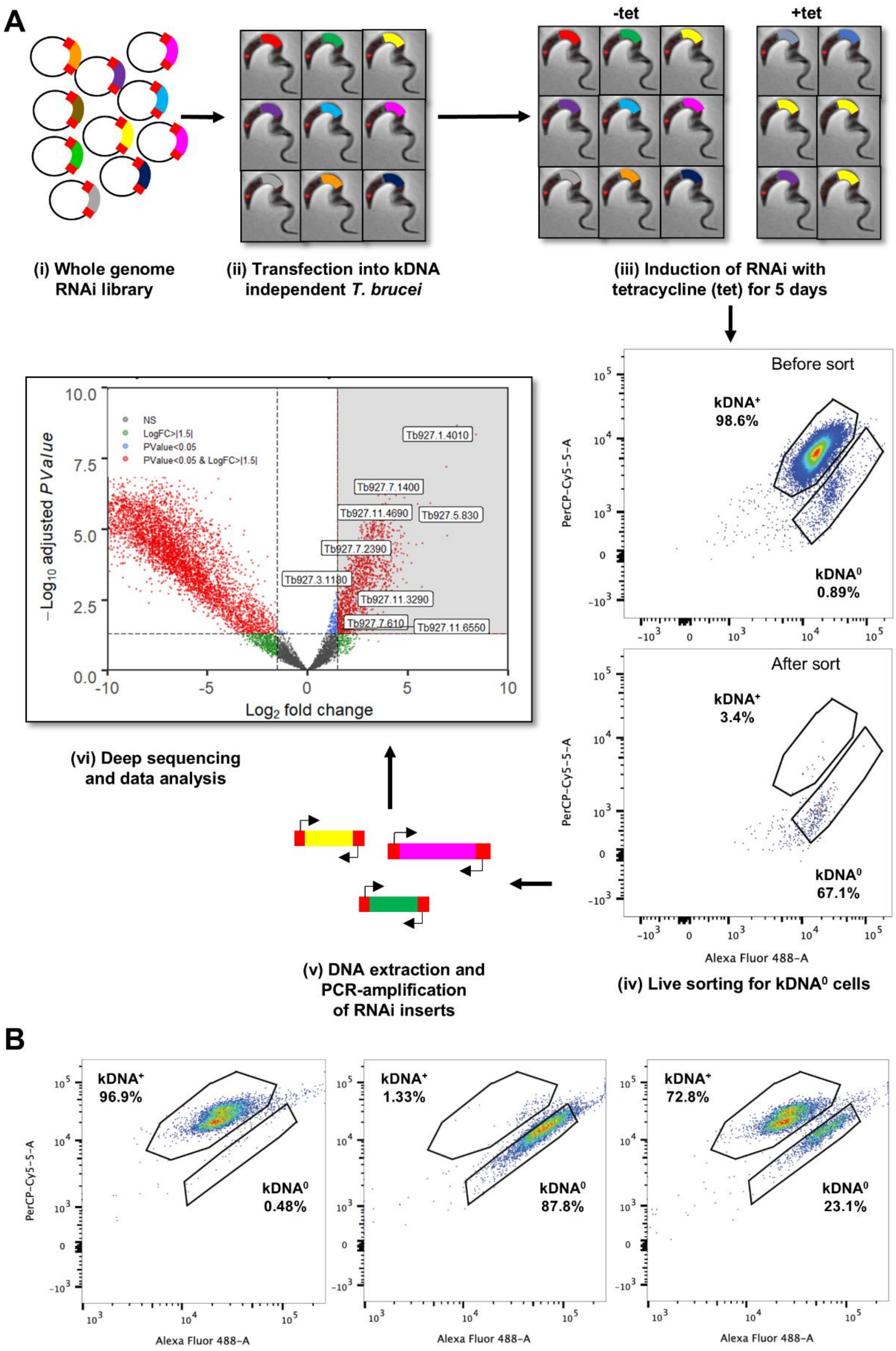
A
-tet
+tet
(i) Whole genome RNAi library
(ii) Transfection into kDNA independent T. brucei
(iii) Induction of RNAi with tetracycline (tet) for 5 days
Before sort
kDNA+ 98.6%
kDNA0 0.89%
After sort
kDNA+ 3.4%
kDNA0 67.1%
PerCP-Cy5-5-A
Alexa Fluor 488-A
(iv) Live sorting for kDNA0 cells
(v) DNA extraction and PCR-amplification of RNAi inserts
(vi) Deep sequencing and data analysis
NS
LogFC>|1.5|
PValue<0.05
PValue<0.05 & LogFC>|1.5|
Tb927.1.4010
Tb927.7.1400
Tb927.11.4690
Tb927.5.830
Tb927.7.2390
Tb927.3.1180
Tb927.11.3290
Tb927.7.610
Tb927.11.6550
−Log10 adjusted PValue
Log2 fold change
B
kDNA+ 96.9%
kDNA0 0.48%
kDNA+ 1.33%
kDNA0 87.8%
kDNA+ 72.8%
kDNA0 23.1%
PerCP-Cy5-5-A
Alexa Fluor 488-A

Supplementary Figure S1. Screening approach. (**A**) Overview of the kDNA RIT-seq experimental design to find genes that are important for kDNA maintenance. (i, ii) A whole genome RNAi library (Alsford et al., Genome Research 2011) was transfected into kDNA-independent *T. brucei*. (iii) Upon RNAi library induction (+tet), fragments corresponding to genes essential for kDNA maintenance are expected to result in the loss of kDNA from the cell. Note that induction of some RNAi fragments will result in a growth defect and depletion of the cells from the population, but in the kDNA-independent background this should not be the case for genes of interest. (iv) To identify these genes, 5 days after induction, live cells are sorted by FACS after staining of DNA with two dyes, dihydro-ethidium (DHE) and dsFLUOR. These dyes differ in their affinities for kDNA vs. nuclear DNA, and measuring fluorescence in the Alexa Fluor 488 and Cy5-5 channels separates kDNA$^+$ and kDNA^0 populations. The representative image shows the population before sorting for one of the induced replicates. Isolated kDNA^0 cells are then scaled up by growing them for 80 hours in culture media before extraction of total DNA. (v) RNAi fragments are PCR-amplified using RNAi cassette–specific primers. (vi) PCR amplicons are then fragmented and Illumina sequenced. After mapping to the reference genome, the number of mapped reads per gene is compared for the following samples: Comparison 1: day 5 +tet, sorted for kDNA^0 (n = 5) vs. uninduced day 0 (n = 5), unsorted. Comparison 2: day 5 +tet, sorted for kDNA^0 vs. day 5 -tet, sorted for kDNA^0 (n = 5). (**B**) Establishing the FACS protocol for differential staining of kDNA$^+$ and kDNA^0 cells. Images show the FACS plots from optimized conditions using 1 µl dsFLUOR dye per 4 ml of cell suspension and 10 µg/ml dihydroethidium (DHE; see Materials and Methods for details). From left to right, cell populations are: control (kDNA$^+$) populations of kDNA-independent *T. brucei*, the same cell line after treatment with ethidium bromide to remove kDNA (kDNA^0), and a 1:1 mix of these populations.

A

	1	2	3	4	5	6	7	8	9	10
1: CFAC1_040016200	100.00	65.48	40.50	41.19	41.04	21.95	22.60	22.16	23.80	22.17
2: LmjF.10.0810	65.48	100.00	42.06	42.19	41.92	19.95	21.57	23.42	23.97	21.98
3: TcBrA4_0019580	40.50	42.06	100.00	57.09	57.63	21.31	21.21	24.32	22.52	23.60
4: Tb927.8.4240	41.19	42.19	57.09	100.00	58.86	20.34	20.54	23.92	22.50	21.63
5: TvY486_0803620	41.04	41.92	57.63	58.86	100.00	22.01	22.03	24.57	23.41	22.65
6: CFAC1_040016100	21.95	19.95	21.31	20.34	22.01	100.00	48.07	33.40	30.54	32.48
7: LmjF.10.0800	22.60	21.57	21.21	20.54	22.03	48.07	100.00	32.38	30.36	31.18
8: TvY486_0803610	22.16	23.42	24.32	23.92	24.57	33.40	32.38	100.00	45.09	43.07
9: Tb927.8.4230	23.80	23.97	22.52	22.50	23.41	30.54	30.36	45.09	100.00	46.71
10: TcBrA4_0019600	22.17	21.98	23.60	21.63	22.65	32.48	31.18	43.07	46.71	100.00

B

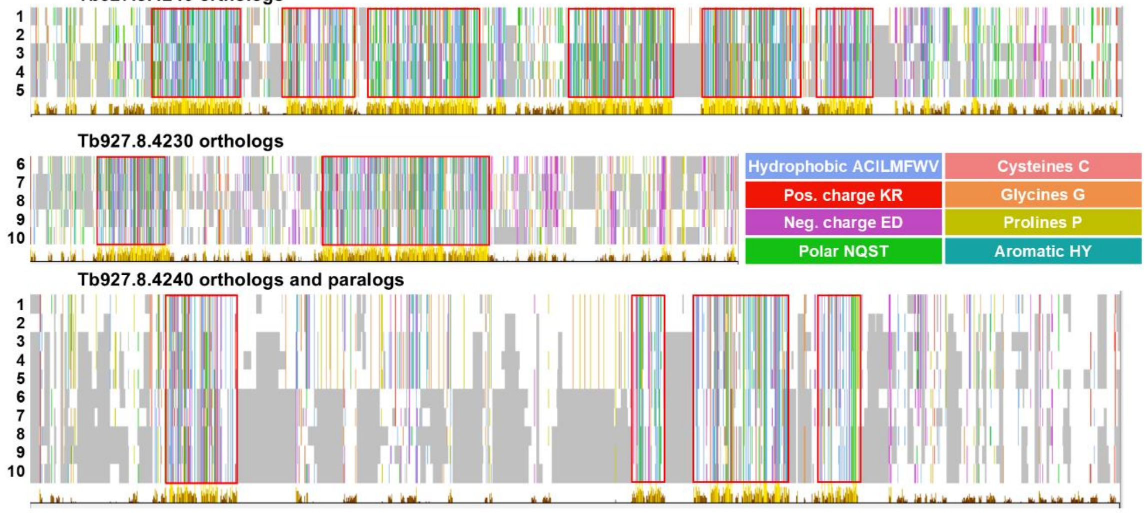

C

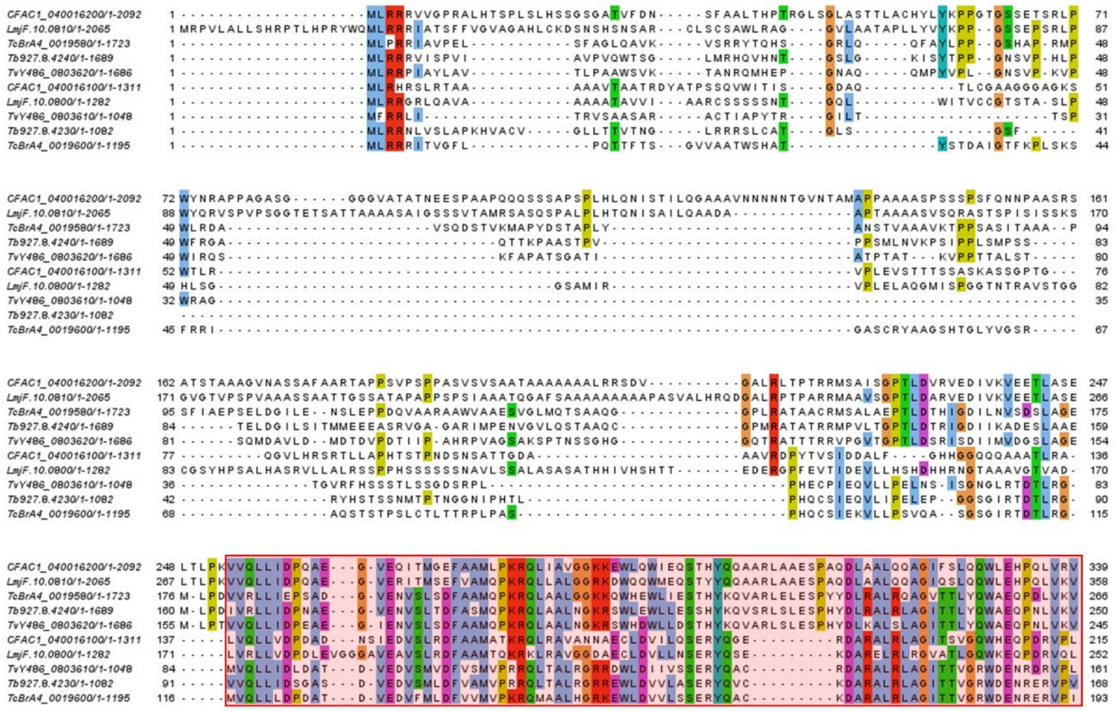

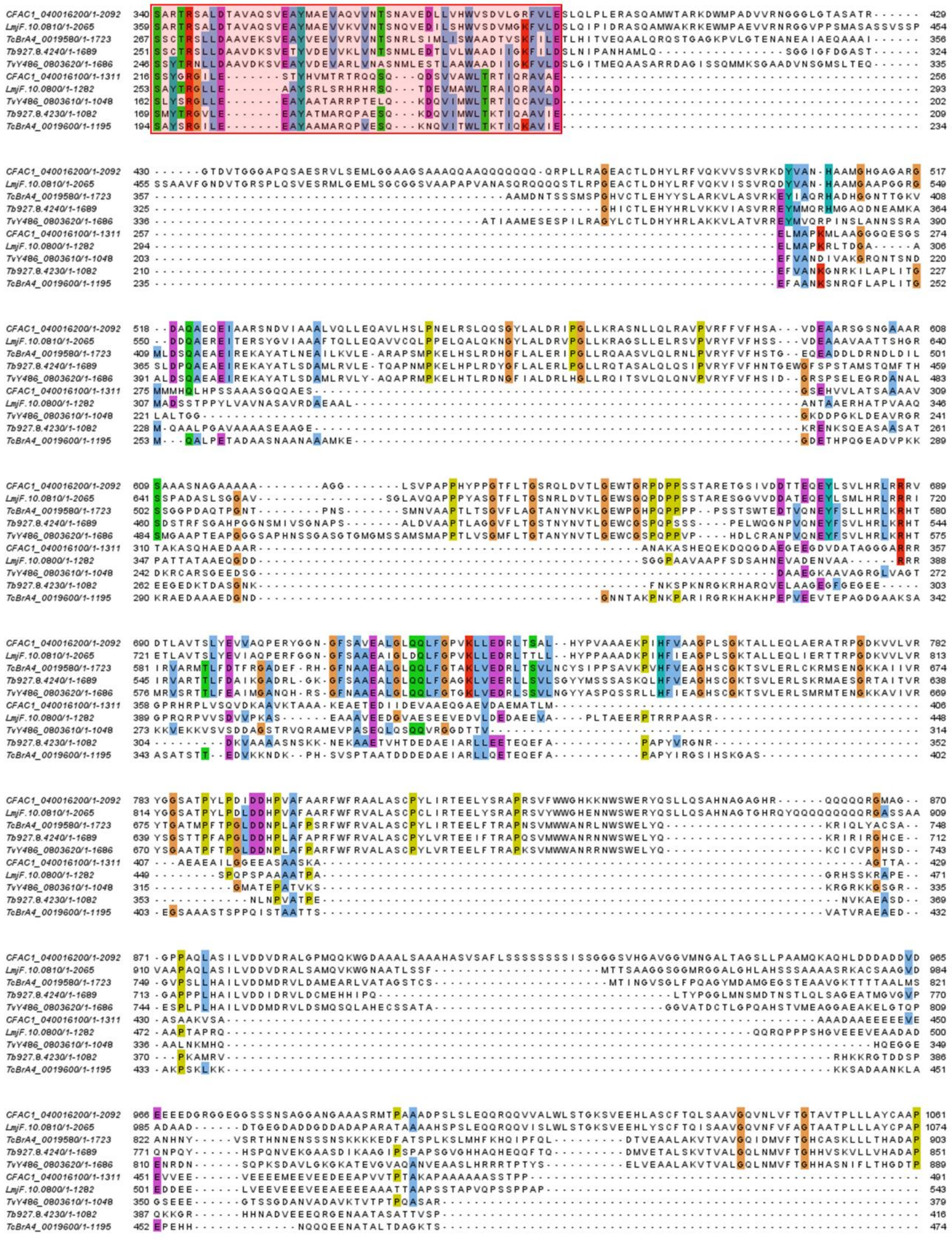

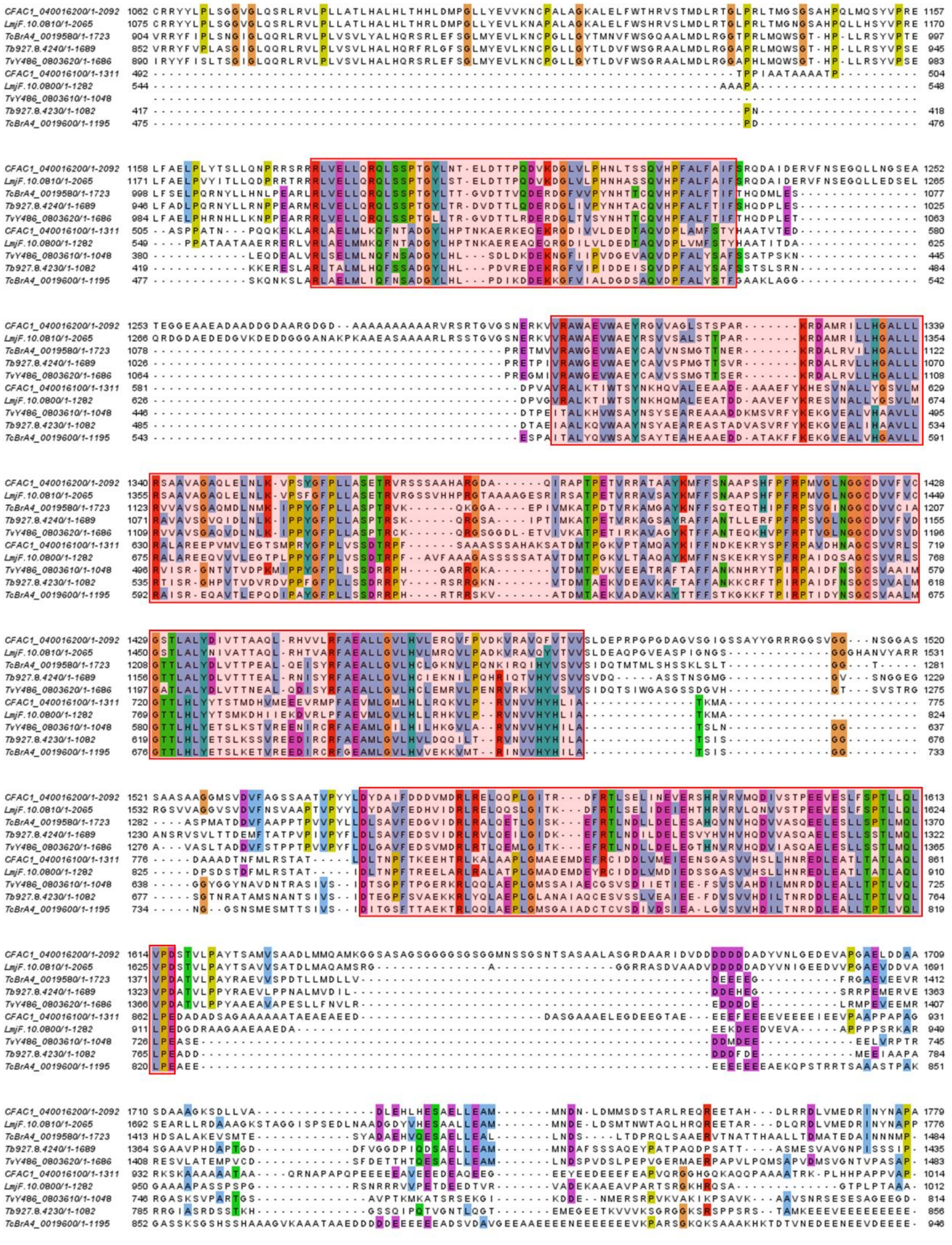
CFAC1_040016200/1-2092
LmjF.10.0810/1-2065
TcBrA4_0019580/1-1723
Tb927.8.4240/1-1689
TvY486_0803620/1-1686
CFAC1_040016100/1-1311
LmjF.10.0800/1-1282
TvY486_0803610/1-1048
Tb927.8.4230/1-1082
TcBrA4_0019600/1-1195

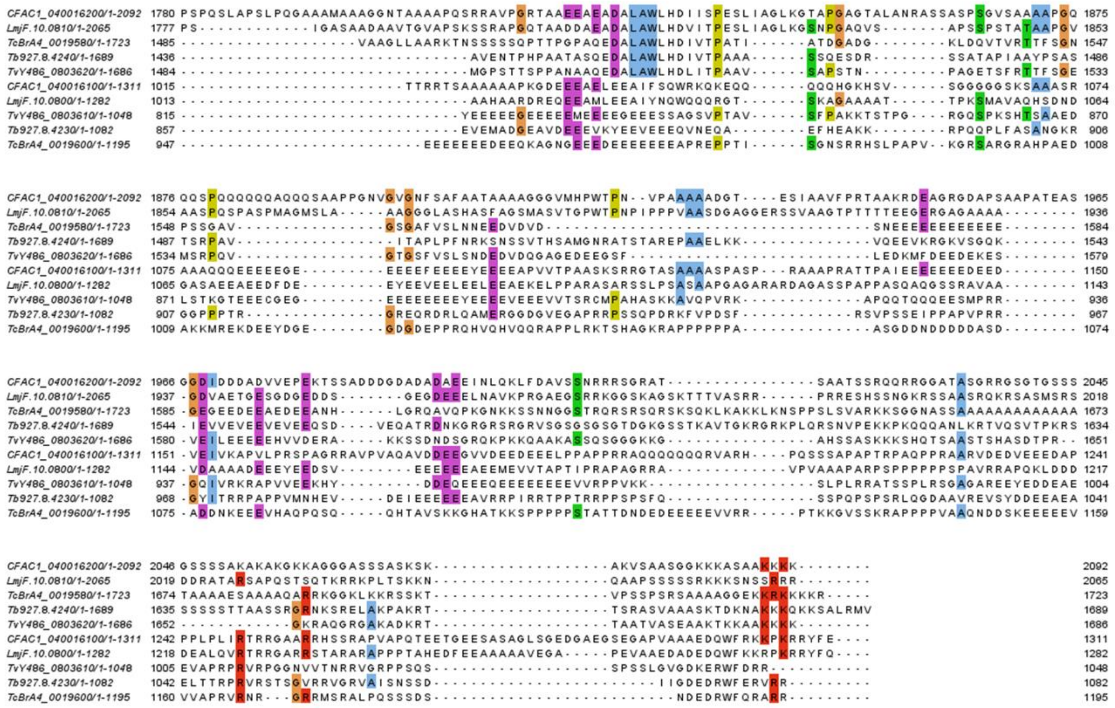

Supplementary Figure S2. Sequence conservation of *Tb*927.8.4240 protein orthologs and paralogs in selected trypanosomatids. Sequences 1-5: *Tb*927.8.4240 orthologs. Sequences 6-10: Tb927.8.4230 orthologs. 1, 6: *Crithidia fasciculata* (Cf-C1). 2, 7: *L. major* (Friedlin). 3, 8: *T. cruzi* (Brazil A4). 4, 9: *T. b. brucei* (TREU 927). 5, 10: *T. vivax* (Y486). (**A**) Percent identity matrix, generated using Clustal 2.1. (**B**) Overviews of sequence conservation in *Tb*927.8.4240 orthologs (top panel), *Tb*927.8.4230 orthologs (middle panel) and *Tb*927.8.4240 orthologs and paralogs combined. Alignments were generated using Muscle (1) and visualized using Jalview (2). Amino acids are colored according to the Clustal color scheme in Jalview. Each residue in the alignment is assigned a color if the amino acid profile of the alignment at that position meets some minimum criteria specific for the residue type (40% identity threshold). Blue: hydrophobic (A, C, I, L, M, F, W, V). Red: positive charge (K, R). Magenta: negative charge (E, D). Green: polar (N, Q, S, T). Pink: cysteines (C). Orange: glycines (G). Yellow: prolines (P). Cyan: aromatic (H, Y). White: unconserved. A track with bars underneath the individual sequences reflects the conservation of the physico-chemical properties for each column of the alignment, calculated based on the AMAS method of multiple sequence alignment analysis (3). The higher the score, the taller the bar and the lighter the shading (highest scores being reflected by yellow). (**C**) Detailed sequence alignment of selected *Tb*927.8.4240 orthologs and paralogs. Sequences and color codes are as in panels A and B. Red boxes and shading indicate regions of relatively high conservation.

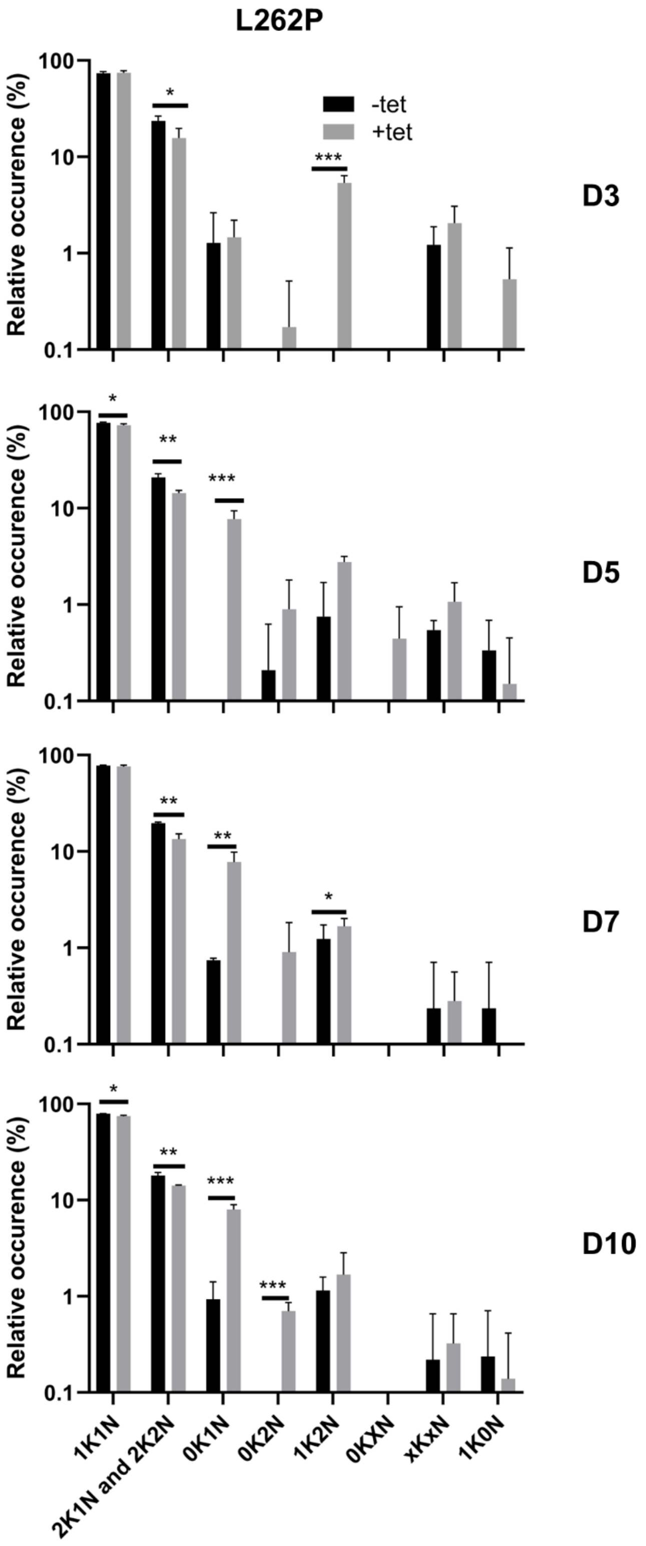

Supplementary Figure S3. Quantification of relative occurrence of kDNA (K) and nuclei (N) in DAPI stained cells from induced (+tet) and non-induced (-tet) *Tb*927.8.4240 RNAi cells in the kDNA-independent γL262P background. Data for days 3, 5, 7 and 10 post-induction are shown ('x' means any value other than 0, 1 or 2; n>100 cells for each triplicate; unpaired t-test P<0.05*, <0.01**, <0.001***).

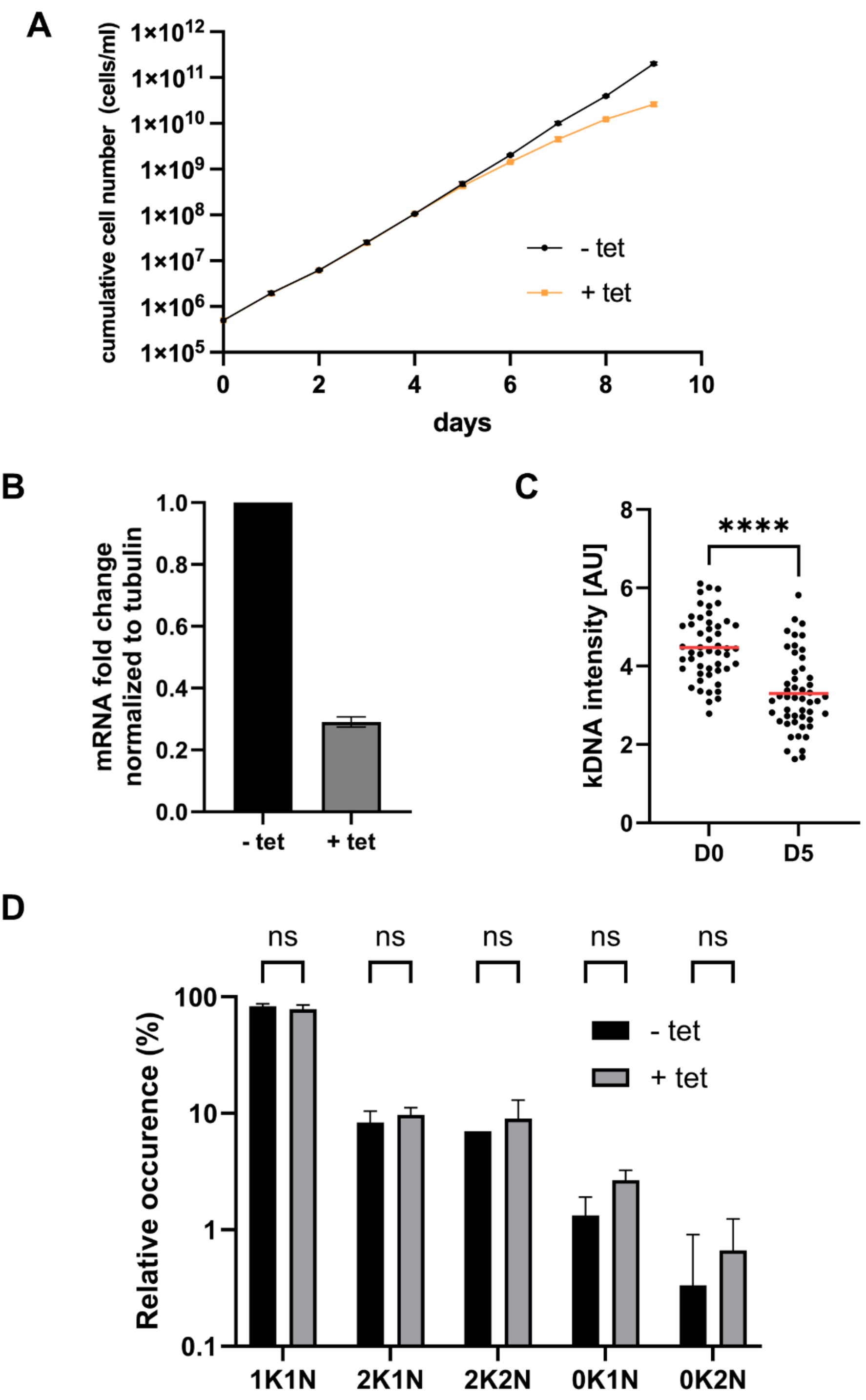

Supplementary Figure S4. Phenotypic analysis of *Tb*927.8.4240 knockdown procyclic trypanosomes. (**A**) Growth curve following RNAi knockdown of *Tb*927.8.4240. The orange line represents induced cells (+tet), and the black line indicates uninduced controls (-tet). Mean values of three replicates. (**B**) *Tb*927.8.4240 mRNA levels measured by quantitative RT-PCR in uninduced and two-day RNAi-induced cells. Mean values of three replicates. (**C**) Quantification of kDNA fluorescence intensity (AU = arbitrary units) in uninduced and five-day RNAi-induced cells (n = 50). Statistical significance was determined using an unpaired t-test (P<0.0001****). (**D**) Quantification of relative occurrence of kDNA (K) and nuclei (N) in DAPI stained cells from non-induced and five-day RNAi-induced cells (100 cells per replicate, three replicates). Statistical analysis was determined using a multiple t-test (ns, not significant).

References

1. Edgar,R.C. (2004) MUSCLE: multiple sequence alignment with high accuracy and high throughput. *Nucleic Acids Res*, **32**, 1792–1797.

2. Waterhouse,A.M., Procter,J.B., Martin,D.M.A., Clamp,M. and Barton,G.J. (2009) Jalview Version 2--a multiple sequence alignment editor and analysis workbench. *Bioinformatics*, **25**, 1189–1191.

3. Livingstone,C.D. and Barton,G.J. (1993) Protein sequence alignments: a strategy for the hierarchical analysis of residue conservation. *Comput Appl Biosci*, **9**, 745–756.
